# m^6^A modification of lncRNA *PCAT6* promotes bone metastasis in prostate cancer through *IGF2BP2*‐mediated *IGF1R* mRNA stabilization

**DOI:** 10.1002/ctm2.426

**Published:** 2021-06-06

**Authors:** Chuandong Lang, Chi Yin, Kaiyuan Lin, Yue Li, Qing Yang, Zhengquan Wu, Hong Du, Dong Ren, Yuhu Dai, Xinsheng Peng

**Affiliations:** ^1^ Department of Orthopaedic Surgery the First Affiliated Hospital Sun Yat‐Sen University Guangzhou China; ^2^ Guangdong Provincial Key Laboratory of Orthopedics and Traumatology Guangzhou China; ^3^ Department of Experimental Research State Key Laboratory of Oncology in South China Collaborative Innovation Center for Cancer Medicine Sun Yat‐sen University Cancer Center Guangzhou China; ^4^ Department of Pathology the First People's Hospital of Guangzhou City Guangzhou China

**Keywords:** bone metastasis, IGF1R, IGF2BP2, PCAT6, prostate cancer

## Abstract

**Background:**

Bone metastasis is the leading cause of tumor‐related death in prostate cancer (PCa) patients. Long noncoding RNAs (lncRNAs) have been well documented to be involved in the progression of multiple cancers. Nevertheless, the role of lncRNAs in PCa bone metastasis remains largely unclear.

**Methods:**

The expression of *prostate cancer‐associated transcripts* was analyzed in published datasets and further verified in clinical samples and cell lines by RT‐qPCR and in situ hybridization assays. Colony formation assay, MTT assay, cell cycle analysis, EdU assay, Transwell migration and invasion assays, wound healing assay, and *in vivo* experiments were carried out to investigate the function of *prostate cancer‐associated transcript 6* (*PCAT6*) in bone metastasis and tumor growth of PCa. Bioinformatic analysis, RNA pull‐down, and RIP assays were conducted to identify the proteins binding to *PCAT6* and the potential targets of *PCAT6*. The therapeutic potential of targeting *PCAT6* by antisense oligonucleotides (ASO) was further explored *in vivo*.

**Results:**

*PCAT6* was upregulated in PCa tissues with bone metastasis and increased *PCAT6* expression predicted poor prognosis in PCa patients. Functional experiments found that *PCAT6* knockdown significantly inhibited PCa cell invasion, migration, and proliferation *in vitro*, as well as bone metastasis and tumor growth *in vivo*. Mechanistically, *METTL3*‐mediated m^6^A modification contributed to *PCAT6* upregulation in an *IGF2BP2*‐dependent manner. Furthermore, *PCAT6* upregulated *IGF1R* expression by enhancing *IGF1R* mRNA stability through the *PCAT6*/*IGF2BP2*/*IGF1R* RNA‐protein three‐dimensional complex. Importantly, *PCAT6* inhibition by ASO *in vivo* showed therapeutic potential against bone metastasis in PCa. Finally, the clinical correlation of *METTL3*, *IGF2BP2*, *IGF1R*, and *PCAT6* was further demonstrated in PCa tissues and cells.

**Conclusions:**

Our study uncovers a novel molecular mechanism by which the m^6^A‐induced *PCAT6*/*IGF2BP2*/*IGF1R* axis promotes PCa bone metastasis and tumor growth, suggesting that *PCAT6* may serve as a promising prognostic marker and therapeutic target against bone‐metastatic PCa.

## Graphical abstract

1. PCAT6 is upregulated in bone metastasis‐positive prostate cancer and PCAT6 upregulation correlates with poor prognosis in patients with prostate cancer.

2. PCAT6 promotes prostate cancer bone metastasis by stabilizing IGF1R mRNA through interacting with IGF2BP2.

3. METTL3‐mediated m^6^A modification leads to the upregulation of PCAT6 in an IGF2BP2‐dependent manner.

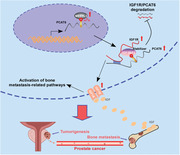

**Abbreviations**
BM: bone metastasis
FISH: fluorescence in situ hybridization
GSEA: gene set enrichment analysis
H&E: hematoxylin and eosin stain
IHC: immunohistochemistry
ISH: in situ hybridization
m^6^A: N6‐methyladenosine
mut: mutant‐type
PCa: prostate cancer
*PCAT6*: prostate cancer‐associated transcript 6
PRAD: prostate adenocarcinoma
RIP: RNA immunoprecipitation
shRNA: small hairpin RNA
TCGA: The Cancer Genome Atlas
Wt: wild‐type

## 1 BACKGROUND

Prostate cancer (PCa) ranks first in terms of incidence and is the second leading cause of cancer‐related mortality in men worldwide.^1^ In clinical practice, metastasis is the foremost cause of death in PCa patients, and bone is the most common distant metastatic organ in PCa.^2^ Appropriately 90% of patients with advanced PCa develop bone metastasis (BM), leading to a decline in quality of life and poor prognosis beyond 5 years.^3^ Unfortunately, the treatment options against BM in PCa remain limited, suggesting that understanding the molecular mechanism of how PCa cells metastasize to the bone will contribute to disease control. Although previous studies have revealed the significant role of proteins and non‐coding RNAs in PCa BM, the precise molecular mechanisms are still largely unclear.

Long noncoding RNAs (lncRNAs) are a class of transcripts more than 200 nt in length with little or no protein‐coding potential, commonly considered transcriptional noise, and their function was unclear for the past two decades. However, with the advancement of biotechnology, many lncRNAs have been identified and shown time‐ and tissue‐specific expression.^4^, ^5^ Simultaneously, dysregulation of lncRNAs has been revealed in many pathological stages, such as tumor growth and metastasis in lung, prostate, gastric, colon, and ovarian cancers.^6^, ^7^, ^8^, ^9^, ^10^ LncRNAs can act as a sponge for miRNAs to inhibit miRNA‐mediated degradation of related target genes, remodel chromatin to modulate the activity of transcriptional regulators, bind to RNA‐binding proteins (RBPs) to control RNA stabilization; or be translated to proteins.^11^, ^12^, ^13^, ^14^ Accumulating emerging findings suggest that lncRNAs may serve as prognostic factors and diagnostic markers, which provide novel insights into the treatment for human diseases, including cancers. However, to the best of our knowledge, studies on the function of lncRNAs in PCa BM are still lacking.

N^6^‐methyladenosine (m^6^A) is the most abundant posttranscriptional modification detected in mRNAs and ncRNAs, that controls RNA fate in several steps, such as RNA splicing and transportation from nucleus to cytoplasm, as well as RNA stabilization and translation efficiency.^15^ In recent years, numerous studies have reported the role of m^6^A‐modified ncRNAs in the progression of human cancers.^15^, ^16^, ^17^, ^18^ For example, m^6^A‐modified *circNSUN2* is transported from nucleus to cytoplasm in a *YTHDC1*‐dependent manner to promote liver metastasis in colorectal cancer.^15^ LncRNA *RP11* which is accumulated in the nucleus induced by m^6^A modification promotes the metastasis of colorectal cancer cells by upregulating *ZEB1*.^19^ METTL14 inhibits the progression of colorectal cancer via degradation of lncRNA *XIST* in an m^6^A‐dependent manner.^20^ Other investigations also revealed that m^6^A modification is required for lncRNA's function and regulates the binding of RBPs to lncRNA.^21^, ^22^ A recent study found that *NEAT1‐1* promotes BM in PCa by enhancing the interaction between *CYCLINL1* and *CDK19* and the Ser2 phosphorylation of *RNPII* through the m^6^A sites in *NEAT1‐1* transcript.^23^ Nevertheless, the role of m^6^A‐modified lncRNA in PCa BM and the underlying mechanisms remain largely unknown, which need to be further investigated.

In this study, we found a specific lncRNA, *prostate cancer‐associated transcript 6* (*PCAT6*), which is located on chromosome 1q32.1. *PCAT6* was significantly upregulated in PCa tissues with BM and related to poor survival in PCa patients. Functional experiments indicated that *PCAT6* knockdown significantly inhibited PCa cell migration, invasion, and proliferation *in vitro*, as well as BM and tumor growth *in vivo*. Mechanistic analysis revealed that *METTL3*‐mediated m^6^A modification of *PCAT6* led to the upregulation of *PCAT6* in an *IGF2BP2*‐dependent manner and *PCAT6* enhanced the stability of *IGF1R* mRNA by interacting with *IGF2BP2*. Clinically, the correlation of the m^6^A/*PCAT6*/*IGF1R* axis with PCa was further verified in PCa tissues and cells. This study suggests that *PCAT6* may function as a crucial marker to predict BM and a promising therapeutic target against bone‐metastatic PCa.

## 2 METHODS

### 2.1 Immunohistochemistry and in situ hybridization assays

Immunohistochemistry (IHC) assay and score calculations were performed following our previous study.^24^ Antibodies against *METTL3* (1:500; #ab195352) and Cytokeratin 8 (CK8; 1:250; #ab53280) from Abcam (Cambridge, UK), *IGF2BP2* (1:300; #11601‐1‐AP) from Proteintech (Wuhan, China), and *IGF1R* (1:2500; #3027), p‐AKT (ser473; 1:100; #4060), Ki67 (1:600; #9449), and p65 (1:1000; #8242) from Cell Signaling Technology (Danvers, MA, USA) were used in the IHC assay to determine their expression in PCa tissues or xenografts tumors. Proliferation index, the percentage of Ki67‐positive tumor cells in all tumor cells, was used to calculate staining score of Ki67. ISH staining and scoring were conducted in accordance with a previous study.^25^ The biotin‐labeled *PCAT6* probe (sequence: 5′‐TCCGCCCCAGTCCAAGCCCAAGGATCCGGTATCGCCTCGACGTCGGGT‐3′, Axl‐bio, Guangzhou, China) was used to assess *PCAT6* expression by ISH staining in paraffin‐embedded PCa tissues. The slides were stained with DAB Enhanced Liquid Substrate System (Sigma, Chicago, IL, USA). The staining score (SI) was assigned by two independent pathologists for comparative evaluation of the expression of target lncRNA or proteins. SI of 4 was used to distinguish tissues with high or low expression of target molecules (high expression: SI > 4; low expression: SI≤4).

### 2.2 Quantitative real‐time PCR

The protocol for quantitative real‐time PCR (RT‐qPCR) was detailly described in a previous study.^26^ Total RNA was extracted from PCa tissues and cells with TRIzol reagent (Invitrogen, Waltham, MA, USA). The primer pairs are presented in Table S1. The relative fold expression was calculated using the comparative threshold cycle (2^−ΔΔCt^ ) method.^27^

### 2.3 Cell proliferation assays

Cell viability was determined by colony formation assay and MTT assay according to a previous study.^3^, ^28^ Cell populations at different phases were detected by cell cycle analysis and EdU assay. Detailed information about the cell cycle analysis is described in our previous study.^27^ EdU assay was performed using EdU Kits (RiboBio, Guangzhou, China) following the manufacturer's instructions.

### 2.4 RNA stability assay

The RNA stability assay was carried out according to the previous description.^16^ Briefly, PCa cells were cultured in 6‐well plates overnight. Next, 5μg/ml actinomycin D (MedChemExpress) was added to PCa cells to inhibit gene transcription for various times as indicated. Then, RNA was extracted and determined by RT‐qPCR. The RNA levels at different times in the indicated group were calculated and normalized to GAPDH.

### 2.5 Tumor model

All animal experiments were approved by the Institutional Animal Care and Use Committee of Sun Yat‐sen University (Approval number: L102012020070J). For the animal model of BM, eight BALB/c‐nu mice (male, 4–6 weeks old) in the indicated groups were injected with PC‐3 cells (1 × 10^6^) in 100 μl PBS into the left cardiac ventricle and further analyzed and measured by In Vivo Imaging System (IVIS, Caliper Life Sciences), X‐ray, hematoxylin and eosin (H&E), and IHC staining as previously described.^2^ To investigate the treatment effect of ASO targeting *PCAT6 in vivo*, 24 mice were used to establish a BM animal model. At 1 week post‐injection with PC‐3 cells, mice were randomly assigned to three groups (*n* = 8 per group): the ASO‐NC group (injection with ASO negative control targeting unknown sequence, 5 nmol in 100 μl PBS for each mouse), the ASO‐L group (injection with low‐dose ASO targeting *PCAT6*, 5 nmol in 100 μl PBS for each mouse), and the ASO‐H group (injection with high‐dose ASO targeting *PCAT6*, 10 nmol in 100 μl PBS for each mouse). ASOs were injected through the tail vein once every 5 days for a total of four times. The tumorigenesis assay was carried out as described previously.^29^ Mice were randomly divided into two groups (male, *n* = 5 per group). The indicated PC‐3 cells (1 × 10^6^) were injected subcutaneously into the left or right dorsum. Tumor volume was measured every 4 days and calculated according to the equation (length × width^2^)/2. At 4 weeks post‐injection, all tumors were harvested and weighed.

### 2.6 Statistical analysis

GraphPad Prism 7.0 and SPSS 19.0 were used for all data analyses. The results are reported as the mean ± standard deviation (SD). *n* represented the number of mice or clinical tissues used in independent experiments. All experiments were repeated three times unless otherwise specified. Significant differences between the two groups were analyzed by Student's *t*‐test for normally distributed data or Mann‐Whitney U test for non‐normally distributed data. For comparison of more than two groups, ANOVA test was used to calculate *p* value. The *χ* ^2^ test was used to compare the rates (constituent ratios) of two groups or analyze the correlation between two categorical variables. For survival analysis, statistical differences between Kaplan‐Meier curves were detected by the log‐rank test. The correlation between two study variables was determined by *Spearman* bivariate correlate analysis. *p* < 0.05 (two‐tailed) was considered statistically significant.

The additional experimental procedures are provided in Supplementary Methods.

## 3 RESULTS

### 3.1 *PCAT6* is upregulated in PCa tissues with BM and related to poor prognosis

**FIGURE 1:**
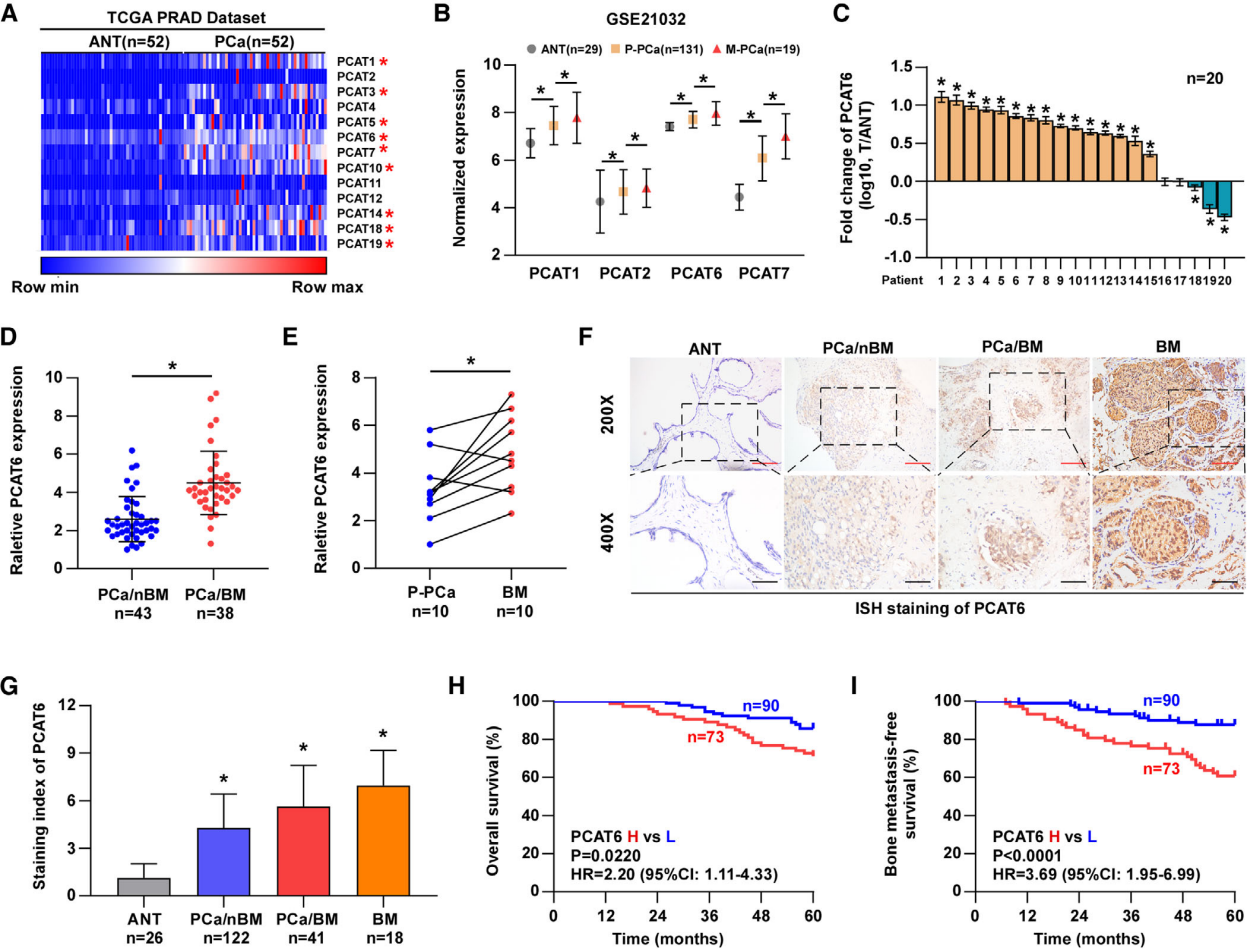
LncRNA *PCAT6* is upregulated in PCa tissues with bone metastasis and related to poor prognosis. (A) The expression pattern of PCa‐associated transcripts (PCATs) in PCa tissues (*n* = 52) and matched adjacent normal tissues (ANT, *n* = 52) in the TCGA‐PRAD dataset. * indicates a significant difference between ANT and PCa. (B) The expression of *PCAT1/2/6/7* in ANT (*n* = 29), primary PCa (P‐PCa, *n* = 131), and metastatic PCa (M‐PCa, *n* = 19) in the GEO dataset (GSE21032). (C) RT‐qPCR analysis of *PCAT6* expression in PCa tissues relative to paired ANT (*n* = 20). Transcript levels were normalized to U6 expression. Individual bar in x‐axis indicates individual patient. Error bars represent the mean ± SD of triplicate experiments. (D) RT‐qPCR analysis of *PCAT6* expression in PCa tissues without bone metastasis (PCa/nBM, *n* = 43) and PCa tissues with bone metastasis (PCa/BM, *n* = 38). Transcript levels were normalized to U6 expression. (E) RT‐qPCR analysis of *PCAT6* expression in 10 pairs of P‐PCa and matched bone metastasis tissues (BM). Transcript levels were normalized to U6 expression. (F) Representative images of ISH analysis of *PCAT6* expression. Scale bar: red, 100 μm; black, 50 μm. (G) ISH analysis of *PCAT6* expression in ANT (*n* = 26), PCa/nBM (*n* = 122), PCa/BM (*n* = 41), and BM (*n* = 18). (H and I) Kaplan–Meier analysis of overall survival (H) and bone metastasis‐free survival (I) curve of PCa patients stratified by *PCAT6* expression. All experiments were performed in biological triplicate. Statistical analyses were performed by unpaired Student's *t*‐test (D), paired Student's *t*‐test (A, C, E), ANOVA test (B, G), and the log‐rank test (H, I). * *p* < 0.05

Emerging evidence suggests that lncRNAs play critical roles in the metastasis of human cancers, but the function of lncRNAs in BM of PCa remains largely unknown. We observed that a class of RNA transcripts associated with PCa (*prostate cancer‐associated transcript*, *PCAT*) have been reported to participate in the pathological process of multiple cancers, such as colorectal, prostate, lung, and liver cancers.^2^, ^30^, ^31^, ^32^, ^33^ Therefore, we wanted to investigate the biological role of *PCATs* in PCa BM. First, we analyzed the lncRNA expression profile in The Cancer Genome Atlas (TCGA) dataset and found that several *PCAT*s were significantly increased in PCa tissues compared with paired adjacent normal prostate tissues (ANT, Figure 1A). Further analysis based on a Gene Expression Omnibus (GEO) dataset (GSE21032) revealed that four *PCAT*s (*PCAT1/2/6/7*) were gradually increased from ANT and primary PCa tissues (P‐PCa) to metastatic PCa tissues (M‐PCa, Figure 1B). Among these dysregulated *PCAT*s, *PCAT1*, *6*, and *7* overlapped in both datasets, which indicated that they may be more likely to participate in the progression of PCa. The function and molecular mechanism of *PCAT1* and *7* have been clarified in PCa in previous studies.^2^, ^31^ However, the biological function and clinical significance of *PCAT6* in PCa progression, particularly BM, remain unclear. Hence, *PCAT6* was selected for further investigation. Then, we evaluated *PCAT6* expression levels in our clinical samples. The results from RT‐qPCR assays showed that *PCAT6* was dramatically upregulated in PCa tissues compared with paired ANT (Figure 1C). The analyses in TCGA dataset also showed a higher expression of *PCAT6* in PCa tissues (*n* = 492) than in normal prostate tissues (*n* = 52; Figure S1a). Since BM is a critical predictive factor for the prognosis of PCa patients and the leading cause of PCa mortality,^24^, ^34^ we further determined whether *PCAT6* expression is associated with BM in PCa. *PCAT6* expression was detected in 43 cases of PCa without BM (PCa/nBM) and 38 cases of PCa with BM (PCa/BM) by RT‐qPCR assay. Our findings demonstrated that the *PCAT6* level was markedly increased in PCa/BM compared with PCa/nBM (Figure 1D). Moreover, we collected primary PCa (P‐PCa) and matched BM tissues from the same patients, and the results showed a prominent upregulation of *PCAT6* in BM compared with P‐PCa (Figure 1E). Further results from in situ hybridization (ISH) assay revealed that the increased *PCAT6* expression was more prevalent in BM and PCa/BM than in PCa/nBM and ANT (Figure 1F and G). We also found that high *PCAT6* expression was positively related to advanced clinicopathological stages and BM status (Table S2). The results from RT‐qPCR analysis suggested that *PCAT6* level was upregulated in PCa cell lines compared to normal prostate cell line (RWPE‐1)(Figure S1b). Kaplan‐Meier analyses based on patient information revealed that high *PCAT6* expression predicted shorter overall and BM‐free survivals (Figure 1H and I). Survival analysis from the TCGA dataset also revealed a positive correlation between high *PCAT6* expression and shorter disease‐free survival (Figure S1c). The above results indicate that *PCAT6* is upregulated in PCa tissues with BM and related to BM and poor prognosis in PCa patients.

### 3.2 *PCAT6* promotes PCa cell migration and invasion *in vitro* and BM *in vivo*

**FIGURE 2:**
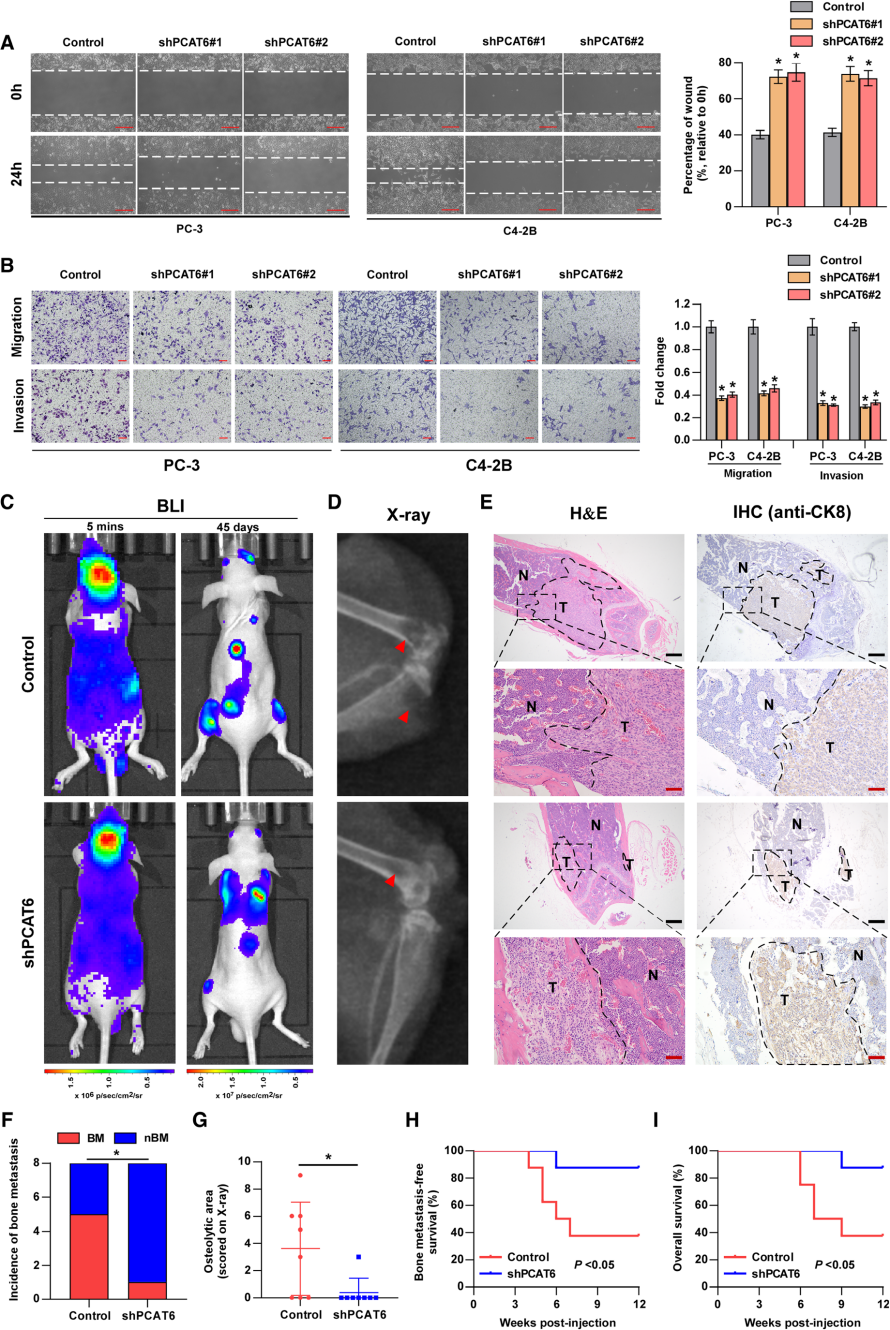
*PCAT6* promotes PCa cell migration and invasion *in vitro* and bone metastasis *in vivo*. (A) Representative images of wound‐healing assays using PC‐3 and C4‐2B cells, showing cell motility after knockdown of *PCAT6* (left panels). Scale bar, 50 μm. Histogram analysis of cell migration distances is shown (right panels). Error bars represent the mean ± SD of triplicate experiments. (B) Representative images of migration and invasion assays using PC‐3 and C4‐2B cells (left panels), showing cell migration and invasion after knockdown of *PCAT6*. Scale bar, 100 μm. Histogram analysis of migrated or invaded cell counts is shown (right panels). Error bars represent the mean ± SD of triplicate experiments. (C) Representative bioluminescent imaging (BLI) of bone metastasis of a mouse from the indicated group at 5 min and 45 days, respectively. (D) Representative radiographic images of bone metastases in the indicated mice (arrows indicate osteolytic lesions). (E) Representative H&E‐stained sections of posterior limbs from the indicated mouse (left panels). Representative IHC staining of bone lesions and tumor lesions was indicated by CK8 staining (right panels). T, tumor; N, the adjacent nontumor tissues; Scar bar: black, 25 μm; red, 5 μm. (F) Incidence of bone metastasis detected in the indicated group (*n* = 8/group; male). (G) The sum of bone metastasis scores for each mouse in the indicated group by X‐ray (*n* = 8/group; male). (H and I) Kaplan–Meier analysis of mouse bone metastasis‐free (H) and overall (I) survival in the indicated groups (*n* = 8/group; male). All experiments were performed in biological triplicate. Statistical analyses were performed by unpaired Student's *t*‐test (A, B), *χ* ^2^ test (F) , Mann–Whitney U test (G) and the log‐rank test (H, I). * *p* < 0.05

To explore the biological function of *PCAT6* in PCa, we performed gene set enrichment analysis (GSEA) based on TCGA data, and the results suggested that *PCAT6* was related to tumor metastasis (Figure S2a). Thus, we explored the role of *PCAT6* in PCa BM. First, *PCAT6* expression was knocked down via lentiviral transfection of two shRNAs in PC‐3 and C4‐2B cells, which express higher *PCAT6* levels among PCa cell lines, and *PCAT6* was overexpressed using exogenous plasmid in 22RV1 cells with lower *PCAT6* levels (Figure S2b). To evaluate the role of *PCAT6* in the metastatic behavior of PCa cells, wound healing and transwell migration and invasion assays were performed. Our data indicated that *PCAT6*‐knockdown cells migrated much more slowly, while *PCAT6*‐overexpressing cells migrated much more quickly than did corresponding control cells (Figure 2A and Figure S2c). Similarly, *PCAT6* silencing dramatically suppressed, while *PCAT6* overexpression promoted PCa cell migration and invasion, as determined by transwell assays (Figure 2B and Figure S2d). In order to exclude the effect of cell proliferation on the results of transwell assays, we used DiD to label PC‐3 cells and performed migration assay. DID is a dye that is distributed equally to each cell during cell division. We found that the migrated cells in the *PCAT6*‐knockdown or control group had the similar intensity of DiD staining within 24 h (Figure S2e), which indicated that there was no significant difference in cell proliferation between the two groups within 24 h. These data suggest that *PCAT6* is a critical regulator of invasion and migration in PCa cells *in vitro*.

To further investigate the effect of *PCAT6* on BM *in vivo*, luciferase‐labeled *PCAT6*‐knockdown or control PC‐3 cells were injected into the left ventricle of BALB/c nude mice to establish a BM animal model. BM status was monitored by the IVIS. The successful injection into the left ventricle was verified by the whole‐body distribution of bioluminescent signals at 5 min post‐injection (Figure 2C). Figure 2C shows a representative image of bioluminescent imaging indicative of BM lesions in mice from the two groups, which were further confirmed by X‐ray (Figure 2D), H&E, and Cytokeratin 8 (CK8, one marker of epithelial cell) staining (Figure 2E). Our data showed a significant decrease in terms of the incidence of BM in the *PCAT6*‐knockdown group relative to the control group (Figure 2F). Further X‐ray analysis indicated that *PCAT6* knockdown markedly inhibited osteolytic lesions of BM (Figure 2G). Survival analysis suggested that high expression of *PCAT6* predicted shorter BM‐free and overall survival (Figure 2H and I). Additionally, *PCAT6* expression was assessed by ISH staining in mice BM tissues. Figure S2f shows decreased *PCAT6* expression in the *PCAT6*‐knockdown group. Overall, *PCAT6* promotes BM of PCa *in vivo*.

### 3.3 *PCAT6* enhances PCa cell proliferation *in vitro* and tumor growth *in vivo*

**FIGURE 3:**
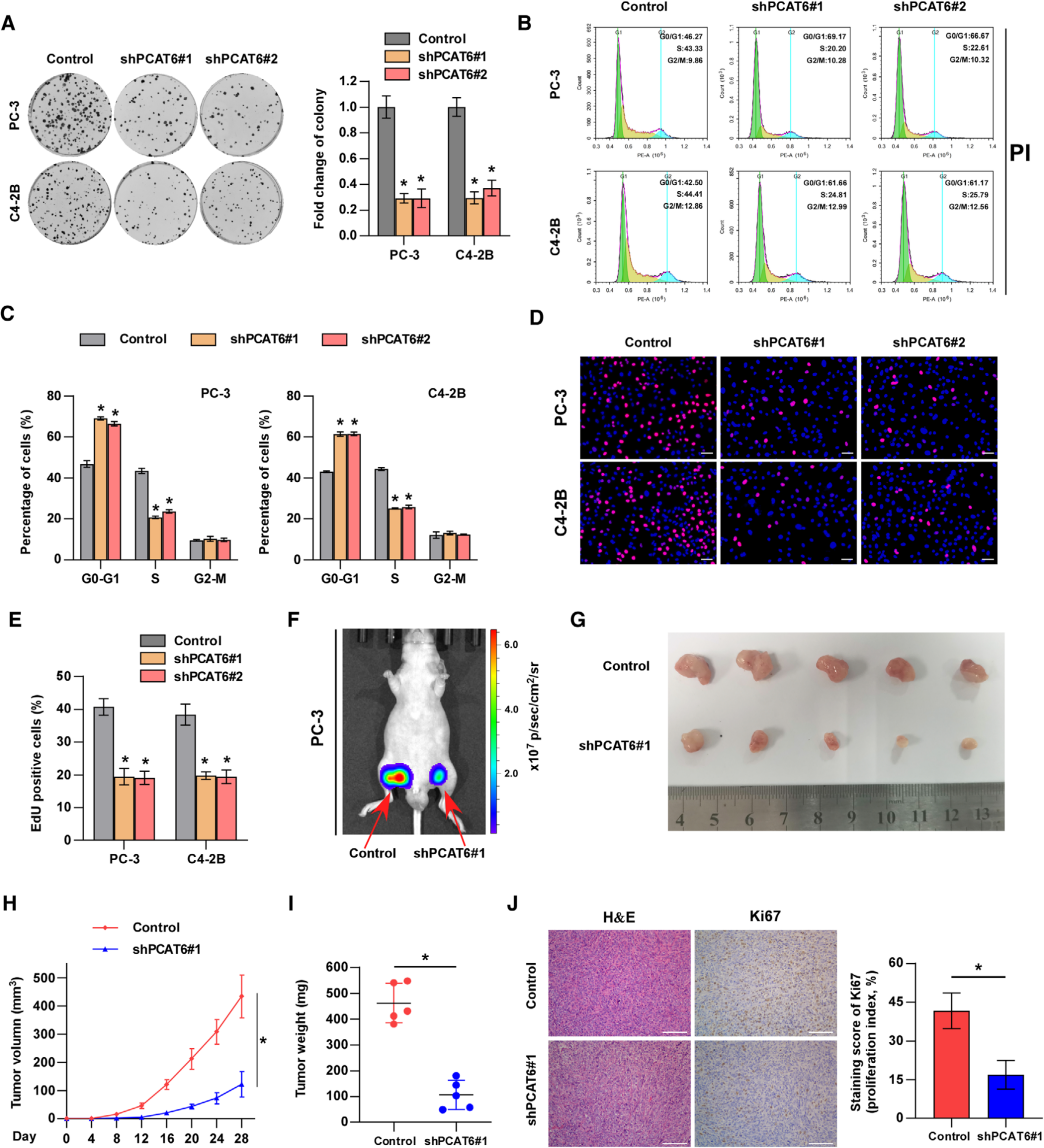
*PCAT6* enhances PCa cell proliferation *in vitro* and tumor growth *in vivo*. (A) Cell viability was evaluated by colony formation assay in *PCAT6*‐control or ‐knockdown PC‐3 and C4‐2B cells (left panels). Histogram analysis of fold change of colony is shown (right panels). Error bars represent the mean ± SD of triplicate experiments. (B–E) Cell populations at different phases were detected by cell cycle analysis (B, C) and EdU (D, E) assays in control or *PCAT6*‐knockdown PC‐3 and C4‐2B cells. PI indicates propidium iodide. Error bars represent the mean ± SD of triplicate experiments. Scale bar, 100 μm. (F) Representative luciferase signal image of the tumor‐bearing mice at 28 days. (G) Tumor sizes in different groups (*n* = 5/group; male). (H) Growth curves of tumors formed by indicated cells (*n* = 5/group; male). (I) Tumor weight in different groups (*n* = 5/group; male). (J) Representative images of H&E staining, IHC staining of Ki67 in mouse tumors (left panels). Histogram analysis of the staining score of Ki67 is shown (right panels). Scale bar, 100 μm. All experiments were performed in biological triplicate. Statistical analyses were performed by unpaired Student's *t*‐test (A, C, E, H, I, J). * *p* < 0.05

The above GSEA also indicated the relationship between *PCAT6* and tumor growth (Figure S3a). Then, the effect of *PCAT6* knockdown on PCa cell proliferation was investigated using MTT and colony formation assays. The results from MTT and colony formation assays indicated that silencing *PCAT6* dramatically inhibited the viability and colony formation ability of PC‐3 and C4‐2B cells, whereas *PCAT6* overexpression had an opposite effect in 22RV1 cells (Figure 3A and Figure S3b and c). Furthermore, we investigated the role of *PCAT6* in the cell cycle by flow cytometry and EdU assays. We found that *PCAT6* silencing significantly decreased the cell population in S phase, whereas it increased the cell population in G0/G1 phase (Figure 3B and C). Similarly, EdU assays showed that knockdown of *PCAT6* prominently reduced the percentage of EdU‐positive cells which are in S phase (Figure 3D and E). Additionally, for cell cycle analysis and EdU assay, *PCAT6* overexpression had an inverse effect in 22RV1 cells (Figure S3d and e). Collectively, these results indicate that *PCAT6* promotes PCa cell proliferation by regulating the transition of G0/G1 to S phase.

To further assess the effect of *PCAT6* on PCa tumor growth *in vivo*, we subcutaneously injected *PCAT6*‐knockdown or control PC‐3 cells into BALB/c nude mice and measured tumor activity. Notably, tumor growth, size, and weight were significantly decreased in the *PCAT6*‐knockdown group compared with the control group (Figure 3F‐I). Moreover, lower expression of Ki67 was observed in the *PCAT6*‐knockdown group than in the control group (Figure 3J). Therefore, these findings suggest that *PCAT6* promotes tumor growth of PCa cells *in vivo*.

### 3.4 *PCAT6* interacts with *IGF2BP2* to play oncogenic roles in PCa

**FIGURE 4:**
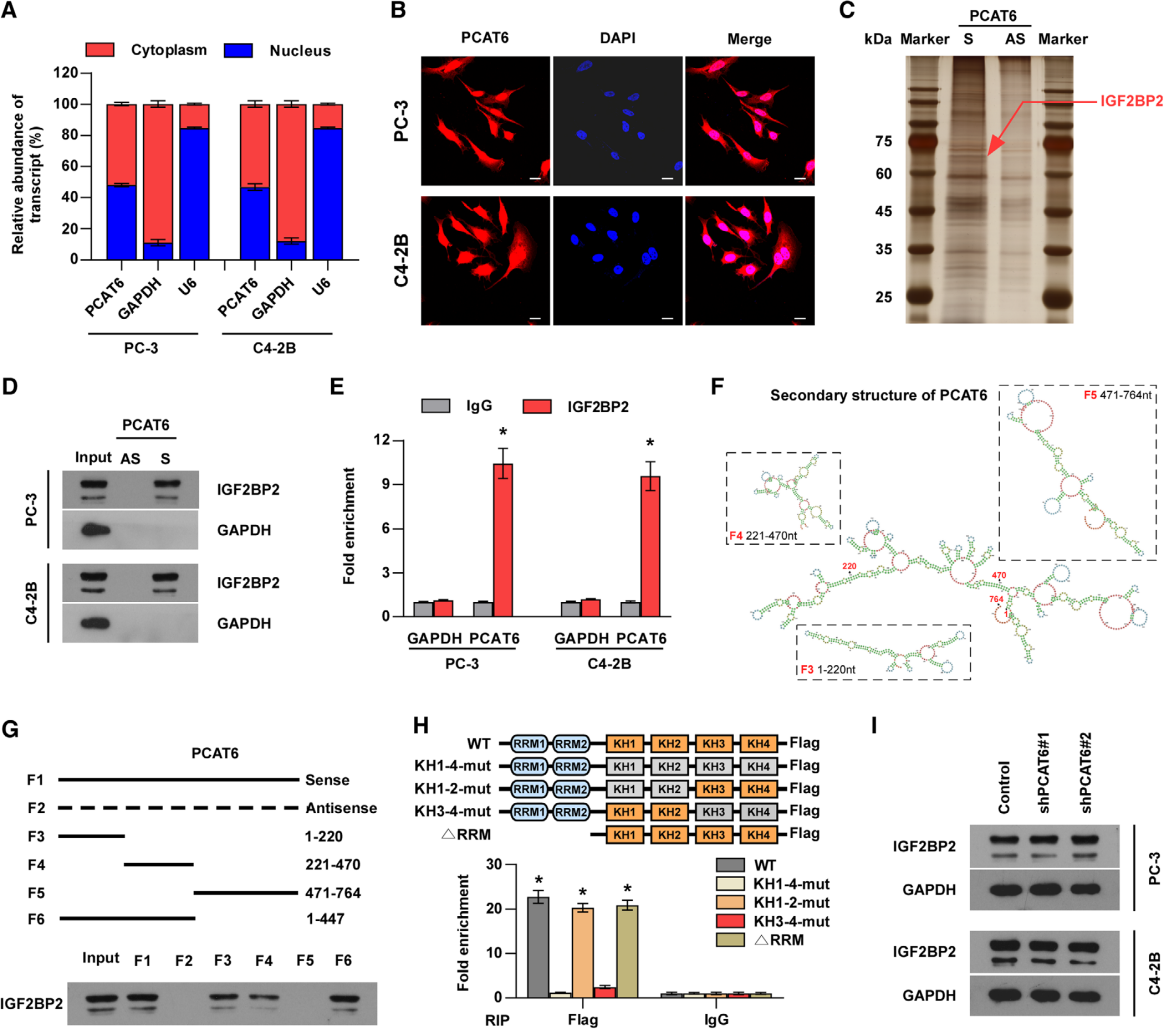
*PCAT6* interacts with *IGF2BP2* to play oncogenic roles in PCa. (A) Nuclear‐cytoplasmic fractionation assays revealing *PCAT6* expression in the cytoplasm and nucleus of PCa cells. U6 and *GAPDH* were used as positive controls in the nucleus and cytoplasm, respectively. Error bars represent the mean ± SD of triplicate experiments. (B) RNA FISH showing the subcellular localization of *PCAT6* in PCa cells. Scar bar, 100 μm. (C) RNA pull‐down assay was performed using *PCAT6* sense and antisense RNAs incubated with cell extracts of PC‐3 cells, followed by silver staining. A red arrow indicates *IGF2BP2*. (D) The interaction between *PCAT6* and *IGF2BP2* was confirmed by RNA pull‐down and western blotting. *GAPDH* served as the negative control. (E) RIP was performed using anti‐*IGF2BP2* and control IgG antibodies, followed by RT‐qPCR to examine the enrichment of *PCAT6* and *GAPDH*. *GAPDH* served as the negative control. Error bars represent the mean ± SD of triplicate experiments. (F) Secondary structure of *PCAT6* predicted by RNAfold Website. (G) Serial deletions of *PCAT6* were used in the RNA pull‐down assays to identify the core regions of *PCAT6* that were required for the physical interaction with *IGF2BP2*. (H) Schematic structures showing RNA‐binding domains within *IGF2BP2* protein and a summary of *IGF2BP2* variants used in this study. Blue boxes are RRM domains, brown boxes are wild‐type KH domains with GxxG core, and gray boxes are inactive KH domains with GxxG to GEEG conversions (upper panels). RIP was performed using anti‐Flag and control IgG antibodies, followed by RT‐qPCR to examine the enrichment of *PCAT6* (down panels). Error bars represent the mean ± SD of triplicate experiments. (I) Western blotting analysis of *IGF2BP2* expression in the indicated group. *GAPDH* served as the loading control. All experiments were performed in biological triplicate. Statistical analyses were performed by *χ* ^2^ test (A) and unpaired Student's *t*‐test (E, H). * *p* < 0.05

The localization of a lncRNA in the cell is closely related to its molecular mechanism.^35^ Subcellular fractionation and RNA‐FISH assays revealed that *PCAT6* was distributed evenly in the nucleus and cytoplasm (Figure 4A and B), suggesting that *PCAT6* can exert its function by various possible mechanisms. Recent reports have demonstrated that lncRNAs may play an oncogenic role in human cancer by interacting with some proteins, particularly RBPs.^35^, ^36^ Hence, we wondered whether *PCAT6* promotes PCa BM by interacting with specific proteins. To identify the *PCAT6*‐binding proteins, we performed RNA pull‐down assays using in vitro‐transcribed biotinylated *PCAT6* and the elution solution was analyzed by mass spectrometry. Meanwhile, to predict the potential proteins interacting with PCAT6, we also analyzed the public databases (StarBase, RPIseq, and catRAPID) and found that seven proteins may interact with PCAT6 (Figure S4a). Notably, only IGF2BP2 (67 kDa) was identified out by both mass spectrometry and public databases and one overtly differential band between 60 and 75 kDa appeared after silver staining (Figure 4C and Figure S4b). Therefore, IGF2BP2 was selected for further investigation. We confirmed the special interaction between *PCAT6* and *IGF2BP2* using western blotting (Figure 4D). RIP experiments also suggested that *PCAT6* was enriched in *IGF2BP2* precipitates (Figure 4E), verifying the above results. To investigate the binding site in *PCAT6*, according to the secondary structure of *PCAT6* predicted by RNAfold, we designed three deletion mutants that mostly preserved the RNA hairpin structures (Figure 4F). The results demonstrated that F3 (1‐220 nt) and F4 (221‐470 nt) of the *PCAT6* transcript bound to *IGF2BP2*, but the binding efficacy was slightly less than full‐length *PCAT6* (Figure 4G). Therefore, we designed F6 (1‐470 nt) and found that F6 bound to *IGF2BP2* as efficiently as full‐length *PCAT6* (Figure 4G), which suggested that F6 (1‐470 nt) was required for the interaction between *PCAT6* and *IGF2BP2*. Next, we studied which domain of *IGF2BP2* mediates the interaction with *PCAT6* and constructed *IGF2BP2* mutants with truncation of the two RRM domains, or with mutations of GxxG to GEEG in the KH domains as reported (Figure 4H).^37^, ^38^ Further RIP assays revealed that the KH3‐4 di‐domain of *IGF2BP2* specifically bound to *PCAT6* (Figure 4H), which indicated that the KH3‐4 domain was indispensable for the interaction between *PCAT6* and *IGF2BP2*. Moreover, *PCAT6* knockdown showed little effect on *IGF2BP2* protein levels in PCa cells (Figure 4I), indicating that the interaction between *PCAT6* and *IGF2BP2* had no effect on the *IGF2BP2* protein itself and *IGF2BP2* was not the downstream target of *PCAT6*.

To further explore the role of *IGF2BP2* in *PCAT6*‐induced BM and proliferation, we overexpressed *IGF2BP2* in *PCAT6*‐knockdown cells (Figure S4c). Notably, *IGF2BP2* overexpression partially reversed *PCAT6* knockdown‐mediated suppression of proliferation and metastasis in PCa cells *in vitro* (Figure S4d‐f). These data indicate that *IGF2BP2* mediates *PCAT6*‐induced BM and proliferation of PCa cells.

### 3.5 *PCAT6*/*IGF2BP2*/*IGF1R* complex stabilizes *IGF1R* mRNA

**FIGURE 5:**
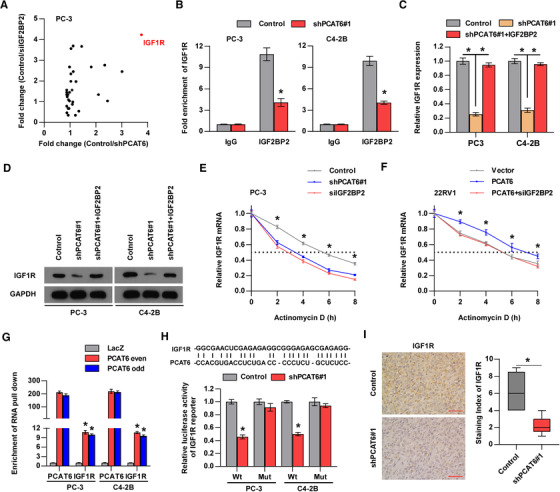
*PCAT6* guides *IGF2BP2* to stabilize *IGF1R* mRNA. (A) Fold change of mRNA expression in control PC‐3 cell relative to *PCAT6*‐ or *IGF2BP2*‐knockdown PC‐3 cell. (B) RIP assays showing the enrichment of *IGF1R* on *IGF2BP2* in control or *PCAT6* knockdown PCa cells. Error bars represent the mean ± SD of triplicate experiments. (C) RT‐qPCR analysis of *IGF1R* expression in the indicated cells. Error bars represent the mean ± SD of triplicate experiments. (D) Western blotting analysis of *IGF1R* expression in the indicated cells. *GAPDH* served as the loading control. (E and F) Control, *PCAT6*‐knockdown, or *IGF2BP2*‐knockdown PC‐3 cells and vector, *PCAT6*‐overexpressing with or without *IGF2BP2*‐knockdown 22RV1 cells were treated with actinomycin D (5 mg/ml) for the indicated periods. Total RNA was purified and then analyzed using RT‐qPCR to examine the mRNA half‐lives of *IGF1R*. Error bars represent the mean ± SD of triplicate experiments. (G) PC‐3 or C4‐2B cell lysates were incubated with in vitro‐synthesized, biotin‐labeled control LacZ DNA probes or antisense DNA probes against *PCAT6* for the biotinylated oligonucleotide pull‐down assay. The precipitates from the pull‐down were analyzed by RT‐qPCR to detect the interacting mRNAs. Error bars represent the mean ± SD of triplicate experiments. (H) The putative *IGF1R*‐binding sites were identified in *PCAT6*. Indicated *IGF1R* wild‐type or mutated *PCAT6* were subjected to luciferase reporter assays in *PCAT6*‐ silenced and control cells. Error bars represent the mean ± SD of triplicate experiments. (I) Representative images of the *IGF1R* expression in xenograft subcutaneous tumor detected by IHC staining (left panels). Histogram showing the staining index in the *PCAT6*‐knockdown and control group (right panels). Scale bar, 100 μm. All experiments were performed in biological triplicate. Statistical analyses were performed by unpaired Student's *t*‐test (B, C, E, F, G, H). * *p* < 0.05

*IGF2BP2* has been reported to modulate the stability of RNAs,^15^, ^16^, ^17^, ^39^ so we wondered whether *PCAT6* guides *IGF2BP2* to stabilize target mRNAs. By analyzing a CLIP‐Seq dataset reported in a previous study,^40^ we found that some mRNAs among the top 100 *IGF2BP2*‐binding mRNAs, such as *C‐MYC*, *AKT3*, *IGF1R*, and *CCND2*, were closely related to tumor progression (Table S3). Therefore, we performed RT‐qPCR assays to identify target mRNAs that were regulated by both *PCAT6* and *IGF2BP2* in these tumor‐related mRNAs and found that *IGF1R* was the most downregulated mRNA both in *PCAT6*‐ and *IGF2BP2*‐knockdown PC‐3 cells compared with control PC‐3 cells (Figure 5A and Figure S5a). Interestingly, previous studies demonstrated that *IGF1R* regulated cancer cell metastasis to bone and tumor growth.^41^, ^42^, ^43^ Therefore, we selected *IGF1R* for further investigation. To explore whether endogenous *IGF2BP2* can bind to *IGF1R* mRNA in PCa cells, RIP assays were carried out, and the results showed the significant enrichment of *IGF1R* mRNA in *IGF2BP2* protein (Figure 5B). Moreover, *PCAT6* knockdown dramatically decreased the interaction of *IGF2BP2* with *IGF1R* (Figure 5B), indicating that the interaction between *IGF2BP2* and *IGF1R* was regulated by *PCAT6*. Furthermore, the results from RT‐qPCR and western blotting analysis showed that *IGF2BP2* overexpression eliminated the downregulation of *IGF1R* expression by *PCAT6* knockdown (Figure 5C and D). We then wondered whether the *PCAT6*/*IGF2BP2* complex regulates *IGF1R* expression by stabilizing *IGF1R* mRNA. To test this hypothesis, PCa cells were treated with actinomycin D to measure the degradation of *IGF1R* mRNA. As shown in Figure 5E and Figure S5b, *PCAT6* or *IGF2BP2* silencing significantly reduced *IGF1R* mRNA stability, which was consistent with our hypothesis. Moreover, our data indicated that *PCAT6* overexpression increased *IGF1R* mRNA stability, whereas *IGF2BP2* knockdown abolished the enhancing effect on *IGF1R* mRNA stability induced by *PCAT6* overexpression (Figure 5F). To further determine whether *PCAT6* directly binds to *IGF1R* mRNA, we performed an RNA pull‐down assay. Our data revealed that endogenous *IGF1R* mRNA coprecipitated with the *PCAT6* transcript in PCa cells (Figure 5G), suggesting an interaction between *PCAT6* and *IGF1R* mRNA. In addition, the base‐pairing analysis showed a putative binding site between *PCAT6* and *IGF1R* (Figure 5H), and we designed the mutant binding site (Figure S5c). Next, luciferase reporter assays were used to verify the binding site and we found that *PCAT6* silencing decreased the luciferase activity of the *IGF1R*‐Wt reporter; whereas the luciferase activity of the *IGF1R*‐Mut reporter remained unchanged with *PCAT6* knockdown (Figure 5H). To investigate whether IGF2BP2 affects the binding between PCAT6 and IGF1R mRNA, RNA pulldown assay was performed and the results indicated that IGF2BP2 knockdown had no effect on the interaction between PCAT6 and IGF1R mRNA (Figure S5d). Finally, IHC analysis in mouse subcutaneous tumors demonstrated that *IGF1R* expression was decreased in the *PCAT6*‐knockdown group compared with the control group, supporting the positive regulation between *PCAT6* and *IGF1R* (Figure 5I). These data suggest that *PCAT6* increases *IGF1R* mRNA stability by forming the *PCAT6*/*IGF2BP2*/*IGF1R* complex, which contributes to *IGF1R* upregulation.

### 3.6 *PCAT6* promotes BM through *IGF1R* signaling in PCa

To further investigate the biological function of *IGF1R* in *PCAT6*‐induced BM and tumor growth, we overexpressed *IGF1R* in *PCAT6*‐silenced PC‐3 and C4‐2B cells (Figure S6a). The results from colony formation and EdU assays demonstrated that *IGF1R* overexpression reversed the inhibitory effect of *PCAT6* knockdown on PCa cell proliferation (Figure S6b and c). Moreover, transwell migration and invasion assays indicated that the upregulation of *IGF1R* abolished the migration and invasion suppression induced by silencing *PCAT6* (Figure S6d and e). Overall, *IGF1R* is critical for the oncogenic role of *PCAT6* in PCa *in vitro*.

**FIGURE 6:**
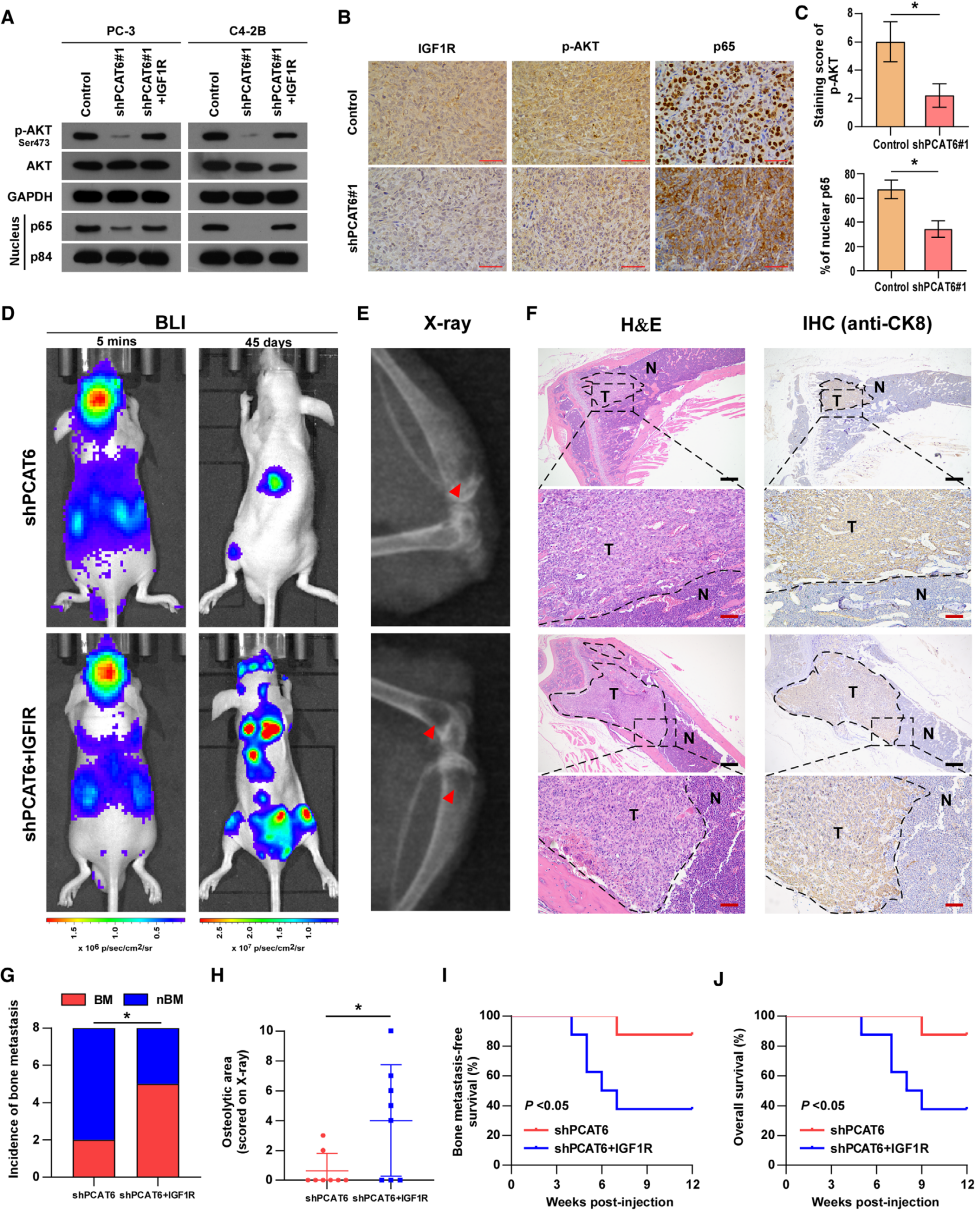
*PCAT6* promotes bone metastasis through *IGF1R* signaling in PCa. (A) Western blotting analysis of p‐AKT(S473), AKT, and p65 expression in the indicated cells. *GAPDH* and P84 served as the loading control. (B) Representative images of *IGF1R*, p‐AKT, and p65 expression in xenograft subcutaneous tumor detected by IHC staining. Scar bar, 100 μm. (C) Histogram showing the staining index in the *PCAT6*‐knockdown and control group. (D) Representative bioluminescent imaging (BLI) of bone metastasis of a mouse from the indicated groups of mice at 5 min and 45 days, respectively. (E) Representative radiographic images of bone metastases in the indicated mice (arrows indicate osteolytic lesions). (F) Representative H&E‐stained sections of posterior limbs from the indicated mouse (left panels). Representative IHC staining of bone lesions and tumor lesions was indicated by CK8 staining (right panels). T, tumor; N, the adjacent nontumor tissues; Scar bar: black, 25 μm; red, 5 μm. (G) Incidence of bone metastasis detected in the indicated group (*n* = 8/group; male). (H) The sum of bone metastasis scores for each mouse in the indicated group by X‐ray (*n* = 8/group; male). (I and J) Kaplan–Meier analysis of mouse bone metastasis‐free (I) and overall (J) survival in the indicated groups (*n* = 8/group; male). All experiments were performed in biological triplicate. Statistical analyses were performed by *χ* ^2^ test (G), Mann–Whitney U test (C, H), and the log‐rank test (I, J). * *p* < 0.05

IGF/*IGF1R* signaling has been reported to be involved in BM of multiple human cancers,^41^, ^43^, ^44^, ^45^, ^46^, ^47^, ^48^ and the PI3K/AKT and NF‐κB pathways are two downstream signaling pathways in IGF/*IGF1R* axis‐induced BM in PCa and breast cancer.^46^ Hence, we performed experiments to determine the role of *IGF1R* in *PCAT6*‐induced BM *in vivo*. First, the effect of *PCAT6* on PI3K/AKT and NF‐κB pathways was investigated. Western blotting revealed that *PCAT6* knockdown significantly suppressed the activity of the PI3K/AKT and NF‐κB pathways and *IGF1R* overexpression attenuated the suppression induced by *PCAT6* knockdown in PCa cells (Figure 6A), which indicated that *IGF1R* mediated the effect of *PCAT6* on PI3K/AKT and NF‐κB pathways. This regulatory relationship was also supported by an IHC assay performed in mouse subcutaneous tumors (Figure 6B and C). Furthermore, as shown in Figure 6D‐J, overexpression of *IGF1R* reversed the suppression of BM by *PCAT6* knockdown *in vivo*, as indicated by the increased incidence and osteolytic lesions, as well as decreased BM‐free and overall survival. Moreover, mice BM tissues were assessed by IHC or ISH assays. Figure S6f shows similar *PCAT6* and IGF2BP2 expression in two groups and increased IGF1R and p‐AKT expression in the group with *PCAT6* knockdown and IGF1R overexpression. Taken together, these findings indicate that *IGF1R* signaling is indispensable for *PCAT6*‐induced BM in PCa.

### 3.7 m^6^A modification contributes to the upregulation of *PCAT6* in PCa

Recent advancements indicate that the epigenetic mechanisms are frequently involved in the dysregulation of lncRNAs, therefore, we wondered whether epigenetic regulation is responsible for *PCAT6* upregulation in PCa. We first treated PCa cells with 5‐aza‐dC, a DNA methyltransferase inhibitor, and found that *PCAT6* expression remained unaltered (Figure S7a), indicating that DNA methylation did not participate in *PCAT6* regulation. The effect of histone acetylation on *PCAT6* expression was determined by treatment with broad‐spectrum HDAC inhibitors (SAHA and NaB) in PC‐3 cells (Figure S7b). The results revealed that histone acetylation was not implicated in the regulation of *PCAT6* in PCa cells.

**FIGURE 7:**
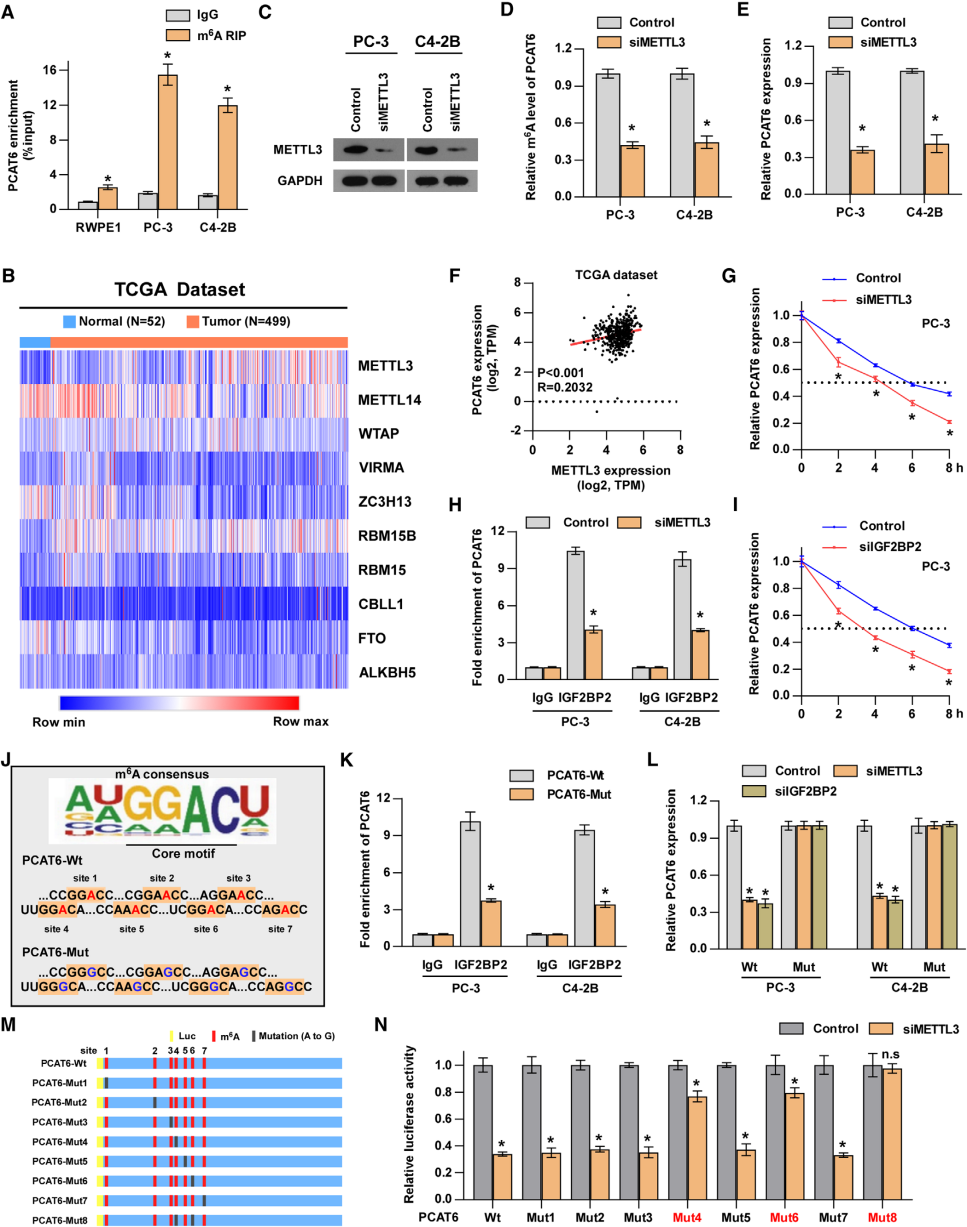
m^6^A modification contributes to the upregulation of *PCAT6* in PCa. (A) m^6^A RIP‐qPCR analysis of *PCAT6* in RWPE‐1, PC‐3, and C4‐2B cells. Error bars represent the mean ± SD of triplicate experiments. (B) Heat map profiling the expression of m^6^A WERs in the TCGA‐PRAD dataset. (C) Western blotting analysis of *METTL3* expression in the indicated cells. *GAPDH* served as the loading control. (D) m^6^A RIP‐qPCR analysis of the m^6^A level in *PCAT6* in the indicated cells. Error bars represent the mean ± SD of triplicate experiments. (E) RT‐qPCR analysis of *PCAT6* expression in the indicated cells. Error bars represent the mean ± SD of triplicate experiments. (F) Correlation analysis showing the correlation between *METTL3* and *PCAT6* in the TCGA‐PRAD dataset. (G) Control or *METTL3*‐knockdown PC‐3 cells were treated with actinomycin D (5 mg/ml) for the indicated periods. Total RNA was purified and then analyzed using RT‐qPCR to examine the mRNA half‐lives of *IGF1R*. Error bars represent the mean ± SD of triplicate experiments. (H) RIP analysis showing the enrichment of *PCAT6* on *IGF2BP2* in the indicated cells. Error bars represent the mean ± SD of triplicate experiments. (I) Control or *IGF2BP2*‐knockdown PC‐3 cells were treated with actinomycin D (5 mg/ml) for the indicated periods. Total RNA was purified and then analyzed using RT‐qPCR to examine the mRNA half‐lives of *IGF1R*. Error bars represent the mean ± SD of triplicate experiments. (J) The putative wild‐type m^6^A sites and designed mutant m^6^A sites in *PCAT6*. (K) RIP analysis showing the enrichment of *PCAT6* on IgG and *IGF2BP2* in the *PCAT6*‐Wt or *PCAT6*‐Mut PCa cells. Error bars represent the mean ± SD of triplicate experiments. (L) RT‐qPCR analysis of *PCAT6* expression in the *PCAT6*‐Wt or *PCAT6*‐Mut PCa cells with or without *METTL3* or *IGF2BP2* knockdown. Transcript levels were normalized to U6 expression. Error bars represent the mean ± SD of triplicate experiments. (M) Schematic representation of mutated (GGAC to GGGC) *PCAT6* of pmirGLO vector to investigate the m^6^A roles on *PCAT6* expression. (N) The luciferase activities of different mutated *PCAT6* reporter in the indicated groups. Error bars represent the mean ± SD of triplicate experiments. All experiments were performed in biological triplicate. Statistical analyses were performed by unpaired Student's *t*‐test (A, D, E, G, H, I, K, L, N) and *Spearman* correlation coefficient (f). * *p* < 0.05

Emerging evidence suggests that m^6^A is the most frequent RNA modification and modulates RNA fate throughout its life cycle, such as processing, translocation, degradation, and translation.^49^, ^50^, ^51^, ^52^ We then investigated the role of m^6^A modification in *PCAT6* upregulation. The results from an online RNA modification website (RMBase, http://rna.sysu.edu.cn/rmbase/) suggested that there are seven m^6^A sites in the *PCAT6* transcript supported by published m^6^A‐ or MeRIP‐Seq data (Figure S7c). Moreover, m^6^A RIP assay showed that m^6^A level was increased in PC‐3 and C4‐2B cells compared with normal prostate cells, RWPE‐1 (Figure 7A), indicating that m^6^A may be involved in *PCAT6* upregulation. To further identify the factor that mediated m^6^A modification in *PCAT6*, the expression pattern of m^6^A‐related genes in PCa was analyzed in the TCGA dataset and the results showed that several genes were dysregulated in PCa (Figure 7B). Then, we verified that *METTL3* was significantly increased in our PCa tissues, while other genes showed no significant differences (Figure S7d). *METTL3*, acting as the key component of the N6‐methyltransferase complex, was also reported to be highly expressed in other human cancers,^16^ which prompted us to investigate its role in regulating m^6^A modification of *PCAT6*. We treated PCa cells with siRNA targeting *METTL3* (Figure 7C) and found that *METTL3* silencing dramatically decreased m^6^A and expression level of *PCAT6* in PCa cells (Figure 7D and E). Intriguingly, there was a positive correlation between *METTL3* and *PCAT6* levels in PCa tissues from the TCGA dataset, supporting the positive regulatory mechanism of *METTL3* on *PCAT6* (Figure 7F); concurrently, *ALKBH5* (the demethylase of m^6^A) overexpression significantly reduced *PCAT6* expression in PCa cells (Figure S7e). The above findings indicated that m^6^A modification was involved in the upregulation of *PCAT6*. To further explore the specific mechanism responsible for the m^6^A‐induced upregulation of *PCAT6* in PCa cells, related assays were performed. The nucleus‐cytoplasm fractionation analysis revealed that *METTL3* knockdown did not affect the localization of *PCAT6* in PCa cells (Figure S7f). Next, we assessed the effect of *METTL3* knockdown on *PCAT6* stability and found a significantly decreased half‐life of *PCAT6* in PCa cells treated with *METTL3* siRNA (Figure 7G), indicating that *METTL3* regulated *PCAT6* expression by modulating *PCAT6* stability. Meanwhile, to investigate whether *METTL3* directly regulates the expression of *PCAT6* gene, we performed some assays. The results shown in Figure S7a and b indicated that *METTL3* cannot regulate the expression of *PCAT6* gene via DNA methylation and histone acetylation. The luciferase reporter assay showed that *METTL3* knockdown did not change the luciferase activity of *PCAT6* promoter (Figure S7g), which suggested that *METTL3* did not participate in the transcriptional regulation of *PCAT6* gene. These data suggest that *METTL3* is not involved in the regulation of *PCAT6* gene. Since m^6^A‐induced regulation of *PCAT6* requires an m^6^A reader to recognize methylated *PCAT6*, we then determined which protein may act as an m^6^A reader to regulate *PCAT6* stability. RIP analysis indicated that *PCAT6* was significantly enriched in *IGF2BP2* protein instead of other m^6^A readers (Figure 7H and Figure S7h) and *METTL3* knockdown dramatically decreased *PCAT6* enrichment in *IGF2BP2* protein (Figure 7H), indicating that *METTL3*‐induced m^6^A modification regulated the recognition and binding of methylated *PCAT6* by *IGF2BP2*. Furthermore, the expression and half‐life of *PCAT6* were strongly reduced upon *IGF2BP2* inhibition in PCa cells (Figure 7I and Figure S7i), which was similar to the results of *METTL3* inhibition. These data suggested that *IGF2BP2* acted as an m^6^A reader for *PCAT6*. In addition, to investigate whether putative m^6^A sites predicted by RMBase are responsible for the interaction between *PCAT6* and *IGF2BP2*, we mutated all putative m^6^A sites, as shown in Figure 7J. Subsequent RIP assays showed that the direct binding between *PCAT6* and *IGF2BP2* was impaired by mutation of the m^6^A sites (Figure 7K). Moreover, *METTL3*‐ or *IGF2BP2*‐mediated regulation of *PCAT6* was abolished after mutation of the m^6^A sites (Figure 7L). To investigate which m^6^A site is responsible for m^6^A‐mediated *PCAT6* stabilization, eight PCAT6 mutants were designed and luciferase reporter assay was performed (Figure 7M). Our results demonstrated that site 4 (‘UUGGACA’) and site 6 (‘UCGGACA’) were involved in regulating *PCAT6* stability (Figure 7N). Furthermore, to investigate whether m^6^A modification affects *PCAT6* secondary structure, we used RNAfold website to predict the secondary structure of *PCAT6*. The prediction results showed that m^6^A sites 4 and 6 in the double strand may block the double strand RNA interaction^22^, ^23^ (Figure S7j), indicating that the sites may exert specific function dependent on different secondary structure. Meanwhile, we found that m^6^A modification had no effect on the interaction between *PCAT6* and *IGF1R* mRNA (Figure S7k), suggesting that this interaction did not rely on m^6^A modification. Additionally, our data also showed that *METTL3* overexpression increased *IGF1R* mRNA expression (Figure S7l). Then, we knocked down *PCAT6* in *METTL3*‐overexpressing cells and found that *PCAT6* knockdown completely reversed *IGF1R* mRNA upregulation induced by *METTL3* overexpression (Figure S7l), which indicated that *METTL3* regulated *IGF1R* expression in a *PCAT6*‐dependent manner. Moreover, previous studies revealed that the direct regulation of *METTL3* on target mRNA requires the interaction between *METTL3* and target mRNA.^53^ To investigate whether *METTL3* also has direct regulation on downstream target *IGF1R*, we performed RIP assay. The results showed that *IGF1R* mRNA was not significantly enriched in *METTL3* immunoprecipitate (Figure S7m), which suggested that *METTL3* may have no direct regulation on *IGF1R*. Overall, the upregulation of *METTL3* promotes m^6^A modification in *PCAT6* and increases *PCAT6* expression in an *IGF2BP2*‐dependent manner.

### 3.8 Targeting *PCAT6* with ASO showed therapeutic potential against PCa BM *in vivo*

**FIGURE 8:**
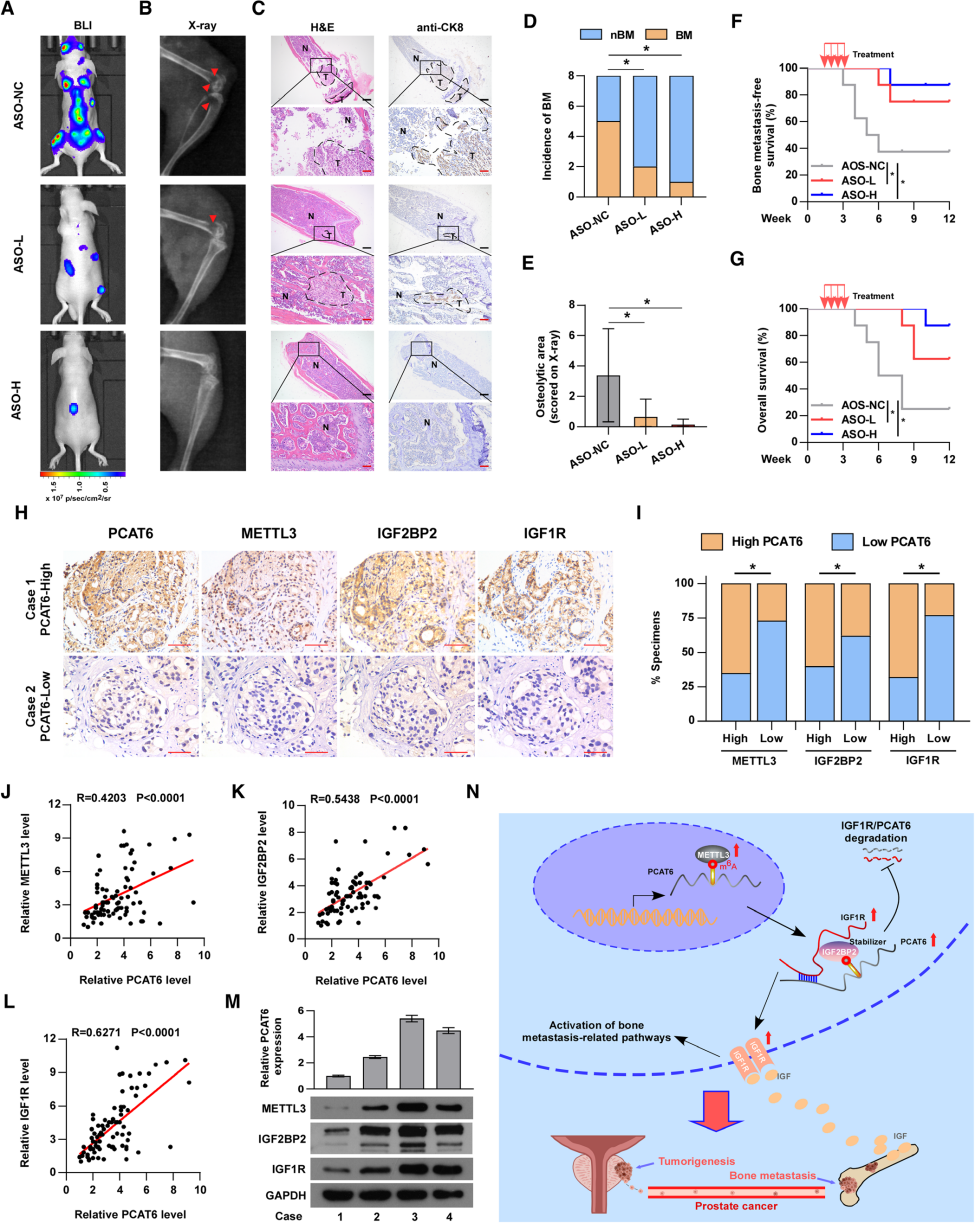
Clinical relevance of m^6^A/*PCAT6*/*IGF1R* axis in PCa. (A) Representative bioluminescent imaging (BLI) of bone metastasis of a mouse from the indicated groups of mice, respectively. (B) Representative radiographic images of bone metastases in the indicated mice (arrows indicate osteolytic lesions). (C) Representative H&E‐stained sections of posterior limbs from the indicated mouse (left panels). Representative IHC staining of bone lesions and tumor lesions were indicated by CK8 staining (right panels). T, tumor; N, the adjacent nontumor tissues; Scar bar: black, 25 μm; red, 5 μm. (D) Incidence of bone metastasis detected in the indicated group (*n* = 8/group; male). (E) The sum of bone metastasis scores for each mouse in the indicated group by X‐ray (*n* = 8/group; male). (f and G) Kaplan–Meier analysis of mouse bone metastasis‐free (F) and overall (G) survival in the indicated group (*n* = 8/group; male). (H) Representative images showing high or low expression of *PCAT6*, *METTL3*, *IGF2BP2*, and *IGF1R* in PCa tumor specimens. Scar bar: 50 μm. (I) Correlation between *PCAT6* and *METTL3 IGF2BP2* or *IGF1R* in 163 PCa tumor specimens. (J–L) Correlation analysis showing the correlation between *PCAT6* and *METTL3* (J), *IGF2BP2* (k), or *IGF1R* (L) in our PCa tissues. (M) RT‐qPCR and western blotting analysis of *PCAT6* and *METTL3*, *IGF2BP2*, or *IGF1R* expression in primary PCa cells reported in our previous study. U6 was used as the control for *PCAT6* loading. *PCAT6* expression levels were normalized to that *PCAT6* expression of case 1. *GAPDH* was served as loading controls. (N) Schematic diagram of the regulatory mechanism of the m^6^A/*PCAT6*/*IGF1R* axis in promoting PCa cell bone metastasis and tumor growth. *METTL3*‐mediated m^6^A modification for *PCAT6* led to the upregulation of *PCAT6* in an *IGF2BP2*‐dependent manner; meanwhile, *PCAT6* enhanced the stability of *IGF1R* mRNA via forming *PCAT6*/*IGF2BP2*/*IGF1R* RNA–protein complex. All experiments were performed in biological triplicate. Statistical analyses were performed by ANOVA test (E), *χ* ^2^ test (D, I), *Spearman* correlation coefficient (J, K, L), and the log‐rank test (F, G). * *p* < 0.05

Recently, ASO drugs have been developed for disease control and preliminarily validated for their capability to target mRNAs and ncRNAs *in vitro* and *vivo*.^54^, ^55^, ^56^ To investigate the therapeutic effect of *PCAT6* interference by ASO, an ASO targeting *PCAT6* was designed for further investigation. First, we found a dose‐dependent inhibition of *PCAT6* by ASO in PCa cells *in vitro* (Figure S8a). To investigate whether ASO targeting *PCAT6* affects m^6^A level of *PCAT6*, m^6^A RIP assay was performed and the results indicated that ASO targeting *PCAT6* had no effect on m^6^A level of *PCAT6* (Figure S8b). Subsequently, a BM model was used to determine the therapeutic efficacy of the ASO targeting *PCAT6 in vivo*. After 1 week of left ventricle injection with wild‐type PC‐3 cells, mice were randomly divided into three groups: ASO‐NC (negative control), ASO‐L (5 nmol ASO), and ASO‐H (10 nmol ASO), and received the corresponding treatment (details shown in the Methods section) through tail vein injection once every 5 days for a total of four injections. As presented in Figure 8A‐E, *PCAT6* inhibition by ASO differentially suppressed BM *in vivo*, as supported by the decreased incidence of BM and the reduced osteolytic area. Moreover, ASO targeting *PCAT6* significantly prolonged overall and BM‐free survivals (Figure 8F and G). These data suggest that targeting *PCAT6* with ASO can be used as a potentially effective therapeutic approach against PCa BM.

### 3.9 Clinical relevance of the m^6^A/*PCAT6*/*IGF1R* axis in PCa

To further investigate the clinical relevance of the m^6^A/*PCAT6*/*IGF1R* axis in PCa, we determined the expression levels of *PCAT6*, *METTL3*, *IGF2BP2*, and *IGF1R* in our PCa tissues. The ISH assay was conducted to detect *PCAT6* expression and the IHC assay was performed to show *METTL3*, *IGF2BP2*, and *IGF1R* expression in our PCa tissues (Figure 8H). The results from IHC and ISH assays demonstrated that *PCAT6* expression was positively correlated with *METTL3*, *IGF2BP2*, and *IGF1R* expression (Figure 8I). RT‐qPCR analysis also showed a positive correlation between *PCAT6* expression and *METTL3*, *IGF2BP2*, or *IGF1R* expression (Figure 8J‐L). Interestingly, western blotting confirmed the positive correlation and regulation between *PCAT6* and *METTL3*, *IGF2BP2* or *IGF1R* in our primary PCa cells reported in a previous study (Figure 8M).^27^ Overall, *METTL3*‐mediated m^6^A modification contributes to the upregulation of *PCAT6* in an *IGF2BP2*‐dependent manner and the *PCAT6*/*IGF2BP2*/*IGF1R* complex further stabilizes *IGF1R* mRNA to activate downstream pathways, which promotes BM and tumor growth in PCa (Figure 8N).

## 4 DISCUSSION

BM frequently contributes to poor prognosis and a decline in quality of life in patients with PCa. Currently, the limited treatment options for BM can only reduce the symptoms to provide comfort for patients and do not prolong the patient's overall survival time.^2^ Therefore, revealing the precise mechanisms underlying BM and developing novel potential therapeutic targets are urgently needed for the prevention and treatment of BM. Emerging evidence has demonstrated that lncRNAs are involved in tumor development.^57^, ^58^, ^59^, ^60^, ^61^, ^62^ Nevertheless, the biological role and mechanism of lncRNAs in PCa BM remain largely undiscovered. As far as we know, *PCAT6* was first systematically assessed in PCa BM. In the current study, we found that *PCAT6* was increased in PCa with BM and predicted poor prognosis. Furthermore, *PCAT6* knockdown inhibited BM and tumor growth of PCa cells. Mechanistically, m^6^A modification of *PCAT6* stabilized *IGF1R* mRNA by forming the *PCAT6*/*IGF2BP2*/*IGF1R* complex, further activating the IGF/*IGF1R* signaling axis.

m^6^A is a posttranscriptional modification in mRNA and ncRNA,^63^, ^64^, ^65^ including lncRNA,^19^, ^66^ and participates in the modulation of RNA fate at various levels.^67^, ^68^ Emerging studies indicated that m^6^A modification of RNAs plays crucial roles in cancer progression.^69^ Wu et al. found that m^6^A‐induced nuclear accumulation of lncRNA *RP11* enhanced colorectal cancer liver metastasis by interacting with *hnRNPA2B1* to increase mRNA degradation of *SIAH1* and *FBXO45*.^19^ The study by Wen et al. reported that lncRNA NEAT1 promoted PCa cells metastasis to bone through m^6^A modification.^23^ Similarly, this study also revealed the function of m^6^A‐modified lncRNA in PCa BM. Our data indicated that m^6^A modification resulted in the upregulation of *PCAT6*. Bioinformatic analysis based on published m^6^A‐ or MeRIP‐Seq data and m^6^A‐RIP assays suggested m^6^A modification in *PCAT6*. Further findings showed that *METTL3* acted as an m^6^A writer for *PCAT6* and that m^6^A‐methylated *PCAT6* was recognized by *IGF2BP2*, a proven m^6^A reader, which has been reported to regulate RNA stability in an m^6^A‐dependent manner.^70^ Hu et al. found that *IGF2BP2* recognized m^6^A‐modified *DANCR* and increased the stability of the *DANCR* transcript, which promoted proliferation and cancer stemness‐like properties in pancreatic cancer.^17^ In colorectal carcinoma, m^6^A‐methylated SOX2 mRNA was recognized by *IGF2BP2*, which suppressed RNA degradation of SOX2.^16^ This study showed that *IGF2BP2* or *METTL3* knockdown significantly decreased the half‐life of *PCAT6* in an m^6^A‐dependent manner, consistent with previous findings. In addition to the effect of m^6^A modification on lncRNA fate, m^6^A modification also regulates the function of lncRNA and RNA‐protein interaction.^52^ LncRNA XIST, regulating gene silencing on the X chromosome, was modified with more than 78 m^6^A residues.^21^ The modified XIST was recognized and bound by YTHDC1, and the interaction led to XIST‐mediated transcriptional silencing.^21^ LncRNA NEAT1, methylated with 4 m^6^A residues, interacted with CYCLINL1 through m^6^A site and promoted the connection of CYCLINL1 and CDK19.^23^ In the meantime, NEAT1 selectively bound to the promoter of RUNX2 and recruited CYCLINL1/CDK19 complex on the promoter of RUNX2 in an m^6^A‐dependent manner, further promoting PCa BM.^23^ Interestingly, one recent investigation found that m^6^A regulated the interaction between HNRNPC and polyU tracts through unwinding double‐strand RNA, which was called RNA‐structural switches.^22^ In our study, decreased m^6^A level or mutation of m^6^A sites reduced the interaction between IGF2BP2 and *PCAT6*. However, whether the RNA‐protein interaction is dependent on RNA‐structural switches remains unclear. The results predicted by the RNAfold website suggested that m^6^A modification may affect the secondary structure of *PCAT6*. Therefore, whether m^6^A modification affect the secondary structure of *PCAT6* and the structure change is indispensable for *PCAT6*‐IGF2BP2 interaction should be investigated by further in‐depth RNA structure analysis.

LncRNAs exert biological roles through various mechanisms related to DNA, RNA, and protein.^4^ The family of prostate cancer‐associated transcript has been reported to play crucial roles in human cancer progression.^2^, ^31^, ^71^, ^72^, ^73^, ^74^, ^75^, ^76^ Emerging evidence has revealed the function of PCAT1, 3, 5, 7, 14, and 19 in PCa.^2^, ^31^, ^72^, ^73^, ^74^, ^77^ Through analyzing public databases, we found that PCAT1, 6, and 7 were dysregulated in multiple databases, indicating that they were more likely to regulate BM of PCa. However, our previous study reported that PCAT7 promoted PCa BM via activation of TGF‐β pathway.^2^ Shang et al found that PCAT1 enhanced PCa progression through interacting with FKBP51 to activate AKT and NF‐κB pathways.^31^ Hence, the function of *PCAT6* in PCa BM was explored in this study. Nonetheless, further study should focus on the potential role of other members of PCAT family in PCa BM. Previous studies showed that *PCAT6* mainly acted as an endogenous competitive RNA by sponging miRNAs to enhance tumor progression.^30^, ^71^, ^78^ *PCAT6* participated in cholangiocarcinoma development by endogenously competing with miR‐330‐5p.^78^ *PCAT6* induced colorectal cancer resistance to 5‐fluorouracil‐based chemotherapy by sponging miR‐204 and activating the HMGA2 pathway.^30^ Nevertheless, *PCAT6* has also been reported to bind to the proteins. Shi et al. found that *PCAT6* could directly interact with *EZH2* and repress *LATS2* transcription in non‐small‐cell lung cancer.^33^ In this study, our results indicated that *PCAT6* directly interacted with *IGF2BP2*. Meanwhile, our findings also revealed the direct interaction between the *PCAT6* transcript and *IGF1R* mRNA. Therefore, *PCAT6* could regulate *IGF1R* mRNA stability through the formation of the *PCAT6*/*IGF2BP2*/*IGF1R* complex. Additionally, the silver staining showed that there were also other proteins with 35, 45, and 60 kDa, which may interact with *PCAT6* and mediate *PCAT6* function. Further investigation should address the role of these proteins in *PCAT6* function. Collectively, these results identify a new mechanism by which *PCAT6* upregulates the stability of *IGF1R* mRNA through interacting with *IGF2BP2*, revealing an important role of lncRNAs in RNA metabolism.

Cancer cell metastasis to bone is a complicated multiple‐stage process and increased invasiveness is one crucial initial stage in tumor metastasis. In our study, *PCAT6* promoted PCa cell invasion and migration by upregulating *IGF1R* expression. *IGF1R* has been revealed to regulate cancer cell invasion and migration in multiple human cancers.^42^, ^43^, ^79^, ^80^, ^81^, ^82^ Sekharam et al. reported that *IGF1R* promoted invasion in colon cancer cells by activating the Akt/Bcl‐x(L) pathway.^42^ Activation of the *IGF1R*/STAT3 signaling axis contributes to enhanced invasion and migration in ovarian cancer.^79^ Additionally, the colonization, survival, and proliferation of cancer cells in the bone are also critical for BM.^46^ *IGF*/*IGF1R* signaling is implicated in cancer cell colonization, survival, and proliferation in bone for multiple cancers, including neuroblastoma, PCa, and breast cancer.^41^, ^42^, ^43^, ^47^ The levels of *IGF‐I* and *‐II* in bone were higher than those in other organs of the human body,^46^ so PCa cells with high expression of *IGF1R* tended to metastasize to bone and developed metastatic bone tumors. PI3K/AKT and NF‐κB signaling are commonly accepted to be two effector pathways that are responsible for IGF/*IGF1R* signaling‐mediated BM in PCa.^83^ This study revealed that PI3K/AKT and NF‐κB signaling were inhibited in *PCAT6*‐knockdown PCa cells and mouse subcutaneous tumors, similar to previous findings. Overall, IGF/*IGF1R* signaling is critical in *PCAT6*‐induced BM of PCa, which suggests that anti‐*IGF1R* antibody^47^ or antisense RNA^43^ may be a potentially effective therapeutic strategy for PCa patients with increased *PCAT6* expression.

Recently, targeting ncRNAs has become a promising therapeutic strategy in the control of diseases, including cancers. Small molecules targeting the secondary structure of oncogenic ncRNA can selectively lead to apoptosis of cancer cells,^84^ and small molecules directly interfering with RNA folding that is related to the disease can act as therapeutic agents to target lncRNA *in vivo*.^85^ Additionally, ASO drugs are receiving increased attention due to their ability to target RNAs. Katsushima et al. reported that ASO targeting lncRNA *TUG1* accompanied by a drug delivery system suppressed the tumorigenesis of glioma *in vivo*.^55^ AZD9150 (ASO targeting *STAT3*) was demonstrated to be effective for treating patients with lymphoma in a phase 1b trial.^56^ In this study, we found that *PCAT6* inhibition by ASO suppressed BM in PCa, which indicated that developing small molecules targeting *PCAT6* may serve as a potential therapeutic strategy against BM in PCa.

## 5 CONCLUSIONS

In summary, this study reveals that m^6^A‐modified *PCAT6* interacts with *IGF2BP2* to stabilize *IGF1R* mRNA, which promotes PCa BM and tumor growth. This study indicates that *PCAT6* may serve as a promising prognostic marker and therapeutic target against bone‐metastatic PCa.

## CONFLICT OF INTEREST

The authors declare that there is no conflict of interest.

## ETHICS APPROVAL AND CONSENT TO PARTICIPATE

The study was approved by the Institutional Review Board of The First Affiliated Hospital of Sun Yat‐sen University. All animal experiments were approved by the Institutional Animal Care and Use Committee of Sun Yat‐sen University Cancer Center.

## AUTHORS' CONTRIBUTIONS

X.P., Y.D., C.L., and D.R. developed ideas and drafted the manuscript. C.L., C.Y., K.L., Y.L., and Z.W. conducted the experiments and contributed to the analysis of data. Q.Y. contributed to the analysis of data. H.D. contributed to the analysis of data and revised the manuscript. All authors contributed to revising the manuscript and approved the final version for publication.

## Supporting information

Supporting information

## Data Availability

The datasets generated and/or analyzed during the current study are available from the corresponding author on reasonable request.

## References

[1] Siegel RL , Miller KD , Jemal A . Cancer statistics, 2018. CA Cancer J Clin. 2018;68:7‐30. 29313949 10.3322/caac.21442

[2] Lang C , Dai Y , Wu Z , et al. SMAD3/SP1 complex‐mediated constitutive active loop between lncRNA PCAT7 and TGF‐β signaling promotes prostate cancer bone metastasis. Mol Oncol. 2020;14:808‐828. 31925912 10.1002/1878-0261.12634 PMC7138406

[3] Ren D , Yang Q , Dai Y , et al. Oncogenic miR‐210‐3p promotes prostate cancer cell EMT and bone metastasis via NF‐kappaB signaling pathway. Mol Cancer. 2017;16:117. 28693582 10.1186/s12943-017-0688-6 PMC5504657

[4] Kopp F , Mendell JT . Functional classification and experimental dissection of long noncoding RNAs. Cell. 2018;172:393‐407. 29373828 10.1016/j.cell.2018.01.011 PMC5978744

[5] Kim J , Piao HL , Kim BJ , et al. Long noncoding RNA MALAT1 suppresses breast cancer metastasis. Nat Genet. 2018;50:1705‐1715. 30349115 10.1038/s41588-018-0252-3 PMC6265076

[6] Yu W , Ding J , He M , et al. Estrogen receptor beta promotes the vasculogenic mimicry (VM) and cell invasion via altering the lncRNA‐MALAT1/miR‐145‐5p/NEDD9 signals in lung cancer. Oncogene. 2019;38:1225‐1238. 30250297 10.1038/s41388-018-0463-1

[7] Parolia A , Venalainen E , Xue H , et al. The long noncoding RNA HORAS5 mediates castration‐resistant prostate cancer survival by activating the androgen receptor transcriptional program. Mol Oncol. 2019;13:1121‐1136. 30776192 10.1002/1878-0261.12471 PMC6487714

[8] Zhang E , He X , Zhang C , et al. A novel long noncoding RNA HOXC‐AS3 mediates tumorigenesis of gastric cancer by binding to YBX1. Genome Biol. 2018;19:154. 30286788 10.1186/s13059-018-1523-0 PMC6172843

[9] Yue B , Liu C , Sun H , et al. A positive feed‐forward loop between LncRNA‐CYTOR and Wnt/beta‐catenin signaling promotes metastasis of colon cancer. Mol Ther. 2018;26:1287‐1298. 29606502 10.1016/j.ymthe.2018.02.024 PMC5993983

[10] Yan H , Li H , Li P , et al. Long noncoding RNA MLK7‐AS1 promotes ovarian cancer cells progression by modulating miR‐375/YAP1 axis. J Exp Clin Cancer Res. 2018;37:237. 30249278 10.1186/s13046-018-0910-4 PMC6154914

[11] Lu S , Zhang J , Lian X , et al. A hidden human proteome encoded by 'non‐coding' genes. Nucleic Acids Res. 2019;47:8111‐8125. 31340039 10.1093/nar/gkz646 PMC6735797

[12] Yari H , Jin L , Teng L , et al. LncRNA REG1CP promotes tumorigenesis through an enhancer complex to recruit FANCJ helicase for REG3A transcription. Nat Commun. 2019;10:5334. 31767869 10.1038/s41467-019-13313-z PMC6877513

[13] Xie M , Ma T , Xue J , et al. The long intergenic non‐protein coding RNA 707 promotes proliferation and metastasis of gastric cancer by interacting with mRNA stabilizing protein HuR. Cancer Lett. 2019;443:67‐79. 30502359 10.1016/j.canlet.2018.11.032

[14] Shan Y , Ma J , Pan Y , Hu J , Liu B , Jia L . LncRNA SNHG7 sponges miR‐216b to promote proliferation and liver metastasis of colorectal cancer through upregulating GALNT1. Cell Death Dis. 2018;9:722. 29915311 10.1038/s41419-018-0759-7 PMC6006356

[15] Chen RX , Chen X , Xia LP , et al. N(6)‐methyladenosine modification of circNSUN2 facilitates cytoplasmic export and stabilizes HMGA2 to promote colorectal liver metastasis. Nat Commun. 2019;10:4695. 31619685 10.1038/s41467-019-12651-2 PMC6795808

[16] Li T , Hu P‐S , Zuo Z , et al. METTL3 facilitates tumor progression via an m6A‐IGF2BP2‐dependent mechanism in colorectal carcinoma. Mol Cancer. 2019;18:112. 31230592 10.1186/s12943-019-1038-7 PMC6589893

[17] Hu X , Peng W‐X , Zhou H , et al. IGF2BP2 regulates DANCR by serving as an N6‐methyladenosine reader. Cell Death Diff. 2019;48:1446. 10.1038/s41418-019-0461-z PMC7244758 31804607

[18] Han J , Wang JZ , Yang X , et al. METTL3 promote tumor proliferation of bladder cancer by accelerating pri‐miR221/222 maturation in m6A‐dependent manner. Mol Cancer. 2019;18:110. 31228940 10.1186/s12943-019-1036-9 PMC6588935

[19] Wu Y , Yang X , Chen Z , et al. m(6)A‐induced lncRNA RP11 triggers the dissemination of colorectal cancer cells via upregulation of Zeb1. Mol Cancer. 2019;18:87. 30979372 10.1186/s12943-019-1014-2 PMC6461827

[20] Yang X , Zhang S , He C , et al. METTL14 suppresses proliferation and metastasis of colorectal cancer by down‐regulating oncogenic long non‐coding RNA XIST. Mol Cancer. 2020;19:46. 32111213 10.1186/s12943-020-1146-4 PMC7047419

[21] Patil DP , Chen CK , Pickering BF , et al. m(6)A RNA methylation promotes XIST‐mediated transcriptional repression. Nature. 2016;537:369‐373. 27602518 10.1038/nature19342 PMC5509218

[22] Liu N , Dai Q , Zheng G , He C , Parisien M , Pan T . N(6)‐methyladenosine‐dependent RNA structural switches regulate RNA–protein interactions. Nature. 2015;518:560‐564. 25719671 10.1038/nature14234 PMC4355918

[23] Wen S , Wei Y , Zen C , Xiong W , Niu Y , Zhao Y . Long non‐coding RNA NEAT1 promotes bone metastasis of prostate cancer through N6‐methyladenosine. Mol Cancer. 2020;19:171. 33308223 10.1186/s12943-020-01293-4 PMC7733260

[24] Yang Q , Lang C , Wu Z , et al. MAZ promotes prostate cancer bone metastasis through transcriptionally activating the KRas‐dependent RalGEFs pathway. J Exp Clin Cancer Res. 2019;38:391. 31488180 10.1186/s13046-019-1374-x PMC6729064

[25] Chen J , Liu A , Wang Z , et al. LINC00173.v1 promotes angiogenesis and progression of lung squamous cell carcinoma by sponging miR‐511‐5p to regulate VEGFA expression. Mol Cancer. 2020;19:98. 32473645 10.1186/s12943-020-01217-2 PMC7260858

[26] Li Q , Ye L , Zhang X , et al. FZD8, a target of p53, promotes bone metastasis in prostate cancer by activating canonical Wnt/beta‐catenin signaling. Cancer Lett. 2017;402:166‐176. 28602974 10.1016/j.canlet.2017.05.029

[27] Ren D , Dai Y , Yang Q , et al. Wnt5a induces and maintains prostate cancer cells dormancy in bone. J Exp Med. 2019;216:428‐449. 30593464 10.1084/jem.20180661 PMC6363426

[28] Chen Z , Chen X , Xie R , et al. DANCR promotes metastasis and proliferation in bladder cancer cells by enhancing IL‐11‐STAT3 signaling and CCND1 expression. Mol Ther. 2019;27:326‐341. 30660488 10.1016/j.ymthe.2018.12.015 PMC6391591

[29] Li Q , Ye L , Guo W , Wang M , Huang S , Peng X . PHF21B overexpression promotes cancer stem cell‐like traits in prostate cancer cells by activating the Wnt/β‐catenin signaling pathway. J Exp Clin Cancer Res. 2017;36:85. 28645312 10.1186/s13046-017-0560-y PMC5481925

[30] Wu H , Zou Q , He H , et al. Long non‐coding RNA PCAT6 targets miR‐204 to modulate the chemoresistance of colorectal cancer cells to 5‐fluorouracil‐based treatment through HMGA2 signaling. Cancer Med. 2019;8:2484‐2495. 30938104 10.1002/cam4.1809 PMC6536993

[31] Shang Z , Yu J , Sun L , et al. LncRNA PCAT1 activates AKT and NF‐kappaB signaling in castration‐resistant prostate cancer by regulating the PHLPP/FKBP51/IKKalpha complex. Nucleic Acids Res. 2019;47:4211‐4225. 30773595 10.1093/nar/gkz108 PMC6486551

[32] Chen S , Chen Y , Qian Q , et al. Gene amplification derived a cancer‐testis long noncoding RNA PCAT6 regulates cell proliferation and migration in hepatocellular carcinoma. Cancer Med. 2019;8:3017‐3025. 30968586 10.1002/cam4.2141 PMC6558594

[33] Shi X , Liu Z , Liu Z , et al. Long noncoding RNA PCAT6 functions as an oncogene by binding to EZH2 and suppressing LATS2 in non‐small‐cell lung cancer. EBioMedicine. 2018;37:177‐187. 30314898 10.1016/j.ebiom.2018.10.004 PMC6286630

[34] Macedo F , Ladeira K , Pinho F , et al. Bone metastases: an overview. Oncol Rev. 2017;11:321. 28584570 10.4081/oncol.2017.321 PMC5444408

[35] Xu Y , Wu W , Han Q , et al. New insights into the interplay between non‐coding RNAs and RNA‐binding protein HnRNPK in regulating cellular functions. Cells. 2019;8:62. 10.3390/cells8010062 PMC6357021 30658384

[36] Wang C , Gu Y , Zhang E , et al. A cancer‐testis non‐coding RNA LIN28B‐AS1 activates driver gene LIN28B by interacting with IGF2BP1 in lung adenocarcinoma. Oncogene. 2019;38:1611‐1624. 30353165 10.1038/s41388-018-0548-x

[37] Hüttelmaier S , Zenklusen D , Lederer M , et al. Spatial regulation of beta‐actin translation by Src‐dependent phosphorylation of ZBP1. Nature. 2005;438:512‐515. 16306994 10.1038/nature04115

[38] Huang H , Weng H , Sun W , et al. Recognition of RNA N(6)‐methyladenosine by IGF2BP proteins enhances mRNA stability and translation. Nat Cell Biol. 2018;20:285‐295. 29476152 10.1038/s41556-018-0045-z PMC5826585

[39] Li Z , Gilbert JA , Zhang Y , et al. An HMGA2‐IGF2BP2 axis regulates myoblast proliferation and myogenesis. Dev Cell. 2012;23:1176‐1188. 23177649 10.1016/j.devcel.2012.10.019 PMC3645921

[40] Hafner M , Landthaler M , Burger L , et al. Transcriptome‐wide identification of RNA‐binding protein and microRNA target sites by PAR‐CLIP. Cell. 2010;141:129‐141. 20371350 10.1016/j.cell.2010.03.009 PMC2861495

[41] van Golen CM , Schwab TS , Kim B , et al. Insulin‐like growth factor‐I receptor expression regulates neuroblastoma metastasis to bone. Cancer Res. 2006;66:6570‐6578. 16818629 10.1158/0008-5472.CAN-05-1448

[42] Sekharam M , Zhao H , Sun M , et al. Insulin‐like growth factor 1 receptor enhances invasion and induces resistance to apoptosis of colon cancer cells through the Akt/Bcl‐x(L) pathway. Cancer Res. 2003;63:7708‐7716. 14633695

[43] Burfeind P , Chernicky CL , Rininsland F , Ilan J , Ilan J . Antisense RNA to the type I insulin‐like growth factor receptor suppresses tumor growth and prevents invasion by rat prostate cancer cells in vivo. Proc Natl Acad Sci U S A. 1996;93:7263‐7268. 8692980 10.1073/pnas.93.14.7263 PMC38971

[44] Rieunier G , Wu X , Macaulay VM , Lee AV , Weyer‐Czernilofsky U , Bogenrieder T . Bad to the bone: the role of the insulin‐like growth factor axis in osseous metastasis. Clin Cancer Res. 2019;25:3479‐3485. 30745299 10.1158/1078-0432.CCR-18-2697

[45] Tandon M , Chen Z , Othman AH , Pratap J . Role of Runx2 in IGF‐1Rβ/Akt‐ and AMPK/Erk‐dependent growth, survival and sensitivity towards metformin in breast cancer bone metastasis. Oncogene. 2016;35:4730‐4740. 26804175 10.1038/onc.2015.518

[46] Hiraga T , Myoui A , Hashimoto N , et al. Bone‐derived IGF mediates crosstalk between bone and breast cancer cells in bony metastases. Cancer Res. 2012;72:4238‐4249. 22738911 10.1158/0008-5472.CAN-11-3061 PMC3438359

[47] Goya M , Si M , Nagai K , et al. Growth inhibition of human prostate cancer cells in human adult bone implanted into nonobese diabetic/severe combined immunodeficient mice by a ligand‐specific antibody to human insulin‐like growth factors. Cancer Res. 2004;64:6252‐6258. 15342412 10.1158/0008-5472.CAN-04-0919

[48] Wu J , Sun H , Li J , et al. Increased survival of patients aged 0–29 years with osteosarcoma: a period analysis, 1984–2013. Cancer Med. 2018;7:3652‐3661. 29992762 10.1002/cam4.1659 PMC6089162

[49] Wang T , Kong S , Tao M , Ju S . The potential role of RNA N6‐methyladenosine in cancer progression. Mol Cancer. 2020;19:88. 32398132 10.1186/s12943-020-01204-7 PMC7216508

[50] Huang H , Weng H , Chen J . m(6)A modification in coding and non‐coding RNAs: roles and therapeutic implications in cancer. Cancer Cell. 2020;37:270‐288. 32183948 10.1016/j.ccell.2020.02.004 PMC7141420

[51] Chen Y , Lin Y , Shu Y , He J , Gao W . Interaction between N(6)‐methyladenosine (m(6)A) modification and noncoding RNAs in cancer. Mol Cancer. 2020;19:94. 32443966 10.1186/s12943-020-01207-4 PMC7243333

[52] Zaccara S , Ries RJ , Jaffrey SR . Reading, writing and erasing mRNA methylation. Nat Rev Mol Cell Biol. 2019;20:608‐624. 31520073 10.1038/s41580-019-0168-5

[53] Zeng C , Huang W , Li Y , Weng H . Roles of METTL3 in cancer: mechanisms and therapeutic targeting. J Hematol Oncol. 2020;13:117. 32854717 10.1186/s13045-020-00951-w PMC7457244

[54] Xiu B , Chi Y , Liu L , et al. LINC02273 drives breast cancer metastasis by epigenetically increasing AGR2 transcription. Mol Cancer. 2019;18:187. 31856843 10.1186/s12943-019-1115-y PMC6921600

[55] Katsushima K , Natsume A , Ohka F , et al. Targeting the Notch‐regulated non‐coding RNA TUG1 for glioma treatment. Nat Commun. 2016;7:13616. 27922002 10.1038/ncomms13616 PMC5150648

[56] Reilley MJ , McCoon P , Cook C , et al. STAT3 antisense oligonucleotide AZD9150 in a subset of patients with heavily pretreated lymphoma: results of a phase 1b trial. J Immunother Cancer. 2018;6:119. 30446007 10.1186/s40425-018-0436-5 PMC6240242

[57] Yang L , Kraft VAN , Pfeiffer S , et al. Nonsense‐mediated decay factor SMG7 sensitizes cells to TNFα‐induced apoptosis via CYLD tumor suppressor and the noncoding oncogene Pvt1. Mol Oncol. 2020;14:2420‐2435. 32602581 10.1002/1878-0261.12754 PMC7530794

[58] Wu Q , Ma J , Wei J , Meng W , Wang Y , Shi M . FOXD1‐AS1 regulates FOXD1 translation and promotes gastric cancer progression and chemoresistance by activating the PI3K/AKT/mTOR pathway. Mol Oncol. 2020;15:299‐316. 32460412 10.1002/1878-0261.12728 PMC7782086

[59] Wang QY , Peng L , Chen Y , et al. Characterization of super‐enhancer‐associated functional lncRNAs acting as ceRNAs in ESCC. Mol Oncol. 2020;14:2203‐2230. 32460441 10.1002/1878-0261.12726 PMC7463357

[60] Sun T , Wu Z , Wang X , et al. LNC942 promoting METTL14‐mediated m(6)A methylation in breast cancer cell proliferation and progression. Oncogene. 2020;39:5358‐5372. 32576970 10.1038/s41388-020-1338-9

[61] Liu W , Liu P , Gao H , Wang X , Yan M . Long non‐coding RNA PGM5‐AS1 promotes epithelial‐mesenchymal transition, invasion and metastasis of osteosarcoma cells by impairing miR‐140‐5p‐mediated FBN1 inhibition. Mol Oncol. 2020;14:2660‐2677. 32412676 10.1002/1878-0261.12711 PMC7530781

[62] Kim SS , Baek GO , Ahn HR , et al. Serum small extracellular vesicle‐derived LINC00853 as a novel diagnostic marker for early hepatocellular carcinoma. Mol Oncol. 2020;14:2646‐2659. 32525601 10.1002/1878-0261.12745 PMC7530776

[63] Ma S , Chen C , Ji X , et al. The interplay between m6A RNA methylation and noncoding RNA in cancer. J Hematol Oncol. 2019;12:121. 31757221 10.1186/s13045-019-0805-7 PMC6874823

[64] Huisman B , Manske G , Carney S , Kalantry S . Functional dissection of the m6A RNA modification. Trends Biochem Sci. 2017;42:85‐86. 28063638 10.1016/j.tibs.2016.12.004 PMC5272839

[65] Dominissini D , Moshitch‐Moshkovitz S , Schwartz S , et al. Topology of the human and mouse m6A RNA methylomes revealed by m6A‐seq. Nature. 2012;485:201‐206. 22575960 10.1038/nature11112

[66] Liu P , Zhang B , Chen Z , et al. m(6)A‐induced lncRNA MALAT1 aggravates renal fibrogenesis in obstructive nephropathy through the miR‐145/FAK pathway. Aging (Albany NY). 2020;12:5280‐5299. 32203053 10.18632/aging.102950 PMC7138587

[67] Cao G , Li HB , Yin Z , Flavell RA . Recent advances in dynamic m6A RNA modification. Open Biol. 2016;6:160003. 27249342 10.1098/rsob.160003 PMC4852458

[68] Zhao BS , He C . Fate by RNA methylation: m6A steers stem cell pluripotency. Genome Biol. 2015;16:43. 25723450 10.1186/s13059-015-0609-1 PMC4336730

[69] Sun T , Wu R , Ming L . The role of m6A RNA methylation in cancer. Biomed Pharmacother. 2019;112:108613. 30784918 10.1016/j.biopha.2019.108613

[70] Yang Y , Hsu PJ , Chen YS , Yang YG . Dynamic transcriptomic m(6)A decoration: writers, erasers, readers and functions in RNA metabolism. Cell Res. 2018;28:616‐624. 29789545 10.1038/s41422-018-0040-8 PMC5993786

[71] Shi R , Wu P , Liu M , Chen B , Cong L . Knockdown of lncRNA PCAT6 enhances radiosensitivity in triple‐negative breast cancer cells by regulating miR‐185‐5p/TPD52 axis. Onco Targets Ther. 2020;13:3025‐3037. 32308433 10.2147/OTT.S237559 PMC7152555

[72] Hua JT , Ahmed M , Guo H , et al. Risk SNP‐mediated promoter‐enhancer switching drives prostate cancer through lncRNA PCAT19. Cell. 2018;174:564‐575 e18. 30033362 10.1016/j.cell.2018.06.014

[73] White NM , Zhao SG , Zhang J , et al. Multi‐institutional analysis shows that low PCAT‐14 expression associates with poor outcomes in prostate cancer. Eur Urol. 2017;71:257‐266. 27460352 10.1016/j.eururo.2016.07.012

[74] Ylipaa A , Kivinummi K , Kohvakka A , et al. Transcriptome sequencing reveals PCAT5 as a novel ERG‐regulated long noncoding RNA in prostate cancer. Cancer Res. 2015;75:4026‐4031. 26282172 10.1158/0008-5472.CAN-15-0217

[75] Prensner JR , Chen W , Iyer MK , et al. PCAT‐1, a long noncoding RNA, regulates BRCA2 and controls homologous recombination in cancer. Cancer Res. 2014;74:1651‐1660. 24473064 10.1158/0008-5472.CAN-13-3159 PMC4009928

[76] Prensner JR , Iyer MK , Balbin OA , et al. Transcriptome sequencing across a prostate cancer cohort identifies PCAT‐1, an unannotated lincRNA implicated in disease progression. Nat Biotechnol. 2011;29:742‐749. 21804560 10.1038/nbt.1914 PMC3152676

[77] Salameh A , Lee AK , Cardo‐Vila M , et al. PRUNE2 is a human prostate cancer suppressor regulated by the intronic long noncoding RNA PCA3. Proc Natl Acad Sci U S A. 2015;112:8403‐8408. 26080435 10.1073/pnas.1507882112 PMC4500257

[78] Xin Y , He X , Zhao W , et al. LncRNA PCAT6 increased cholangiocarcinoma cell proliferation and invasion via modulating miR‐330‐5p. Am J Transl Res. 2019;11:6185‐6195. 31632586 PMC6789233

[79] Chen C , Gupta P , Parashar D , et al. ERBB3‐induced furin promotes the progression and metastasis of ovarian cancer via the IGF1R/STAT3 signaling axis. Oncogene. 2020;39:2921‐2933. 32029900 10.1038/s41388-020-1194-7 PMC7346970

[80] Xu L , Zhou R , Yuan L , et al. IGF1/IGF1R/STAT3 signaling‐inducible IFITM2 promotes gastric cancer growth and metastasis. Cancer Lett. 2017;393:76‐85. 28223169 10.1016/j.canlet.2017.02.014

[81] Zhao X , Dou W , He L , et al. MicroRNA‐7 functions as an anti‐metastatic microRNA in gastric cancer by targeting insulin‐like growth factor‐1 receptor. Oncogene. 2013;32:1363‐1372. 22614005 10.1038/onc.2012.156

[82] Sachdev D , Zhang X , Matise I , Gaillard‐Kelly M , Yee D . The type I insulin‐like growth factor receptor regulates cancer metastasis independently of primary tumor growth by promoting invasion and survival. Oncogene. 2010;29:251‐262. 19838209 10.1038/onc.2009.316 PMC2843625

[83] Rieunier G , Wu X , Macaulay VM , Lee AV , Weyer‐Czernilofsky U , Bogenrieder T . Bad to the bone: the role of the insulin‐like growth factor axis in osseous metastasis. Clin Cancer Res. 2019;25:3479‐3485. 30745299 10.1158/1078-0432.CCR-18-2697

[84] Velagapudi SP , Cameron MD , Haga CL , et al. Design of a small molecule against an oncogenic noncoding RNA. Proc Natl Acad Sci U S A. 2016;113:5898‐5903. 27170187 10.1073/pnas.1523975113 PMC4889373

[85] Disney MD , Angelbello AJ . Rational design of small molecules targeting oncogenic noncoding RNAs from sequence. Acc Chem Res. 2016;49:2698‐2704. 27993012 10.1021/acs.accounts.6b00326 PMC5286924

