# Supplementary material for: m6A modification of lncRNA PCAT6 promotes bone metastasis in prostate cancer through IGF2BP2‐mediated IGF1R mRNA stabilization
Source: Clin Transl Med. 2021 Jun 6;11(6):e426. doi: 10.1002/ctm2.426 (PMC8181202; doi:10.1002/ctm2.426)
Supplement: Supplementary file 1 — Supporting information [file CTM2-11-e426-s001.docx]

**Supplementary Figure and Figure Legend**


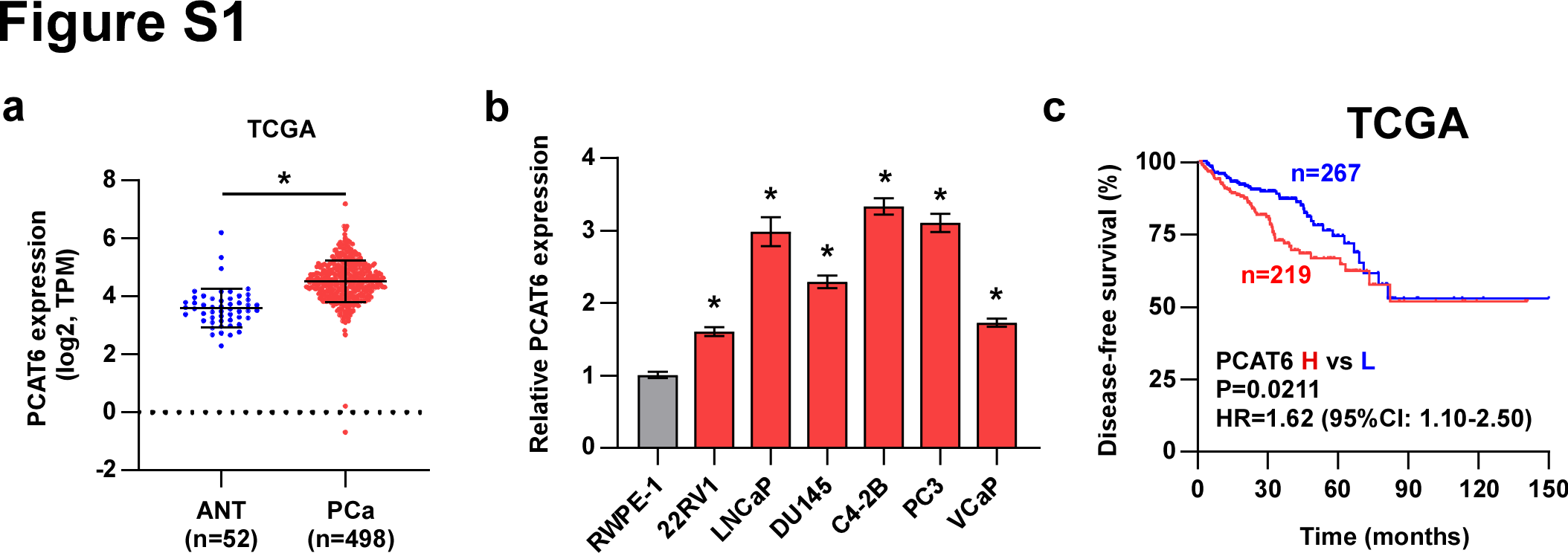


**Figure S1. PCAT6 is upregulated in PCa tissues with bone metastasis and related to poor prognosis.**

**(a)** PCAT6 expression in ANT (n=52) and PCa (n=498) in TCGA dataset. **(b)** RT-qPCR analysis of PCAT6 expression levels in normal prostate epithelial cell (RWPE-1), primary PCa cell 22RV1, brain metastatic cell line DU145, lymph node metastatic cell line LNCaP and three bone metastatic PCa cell lines (PC-3, C4-2B and VCaP). Transcript levels were normalized to U6 expression. Error bars represent the mean ± SD of triplicate experiments. **(c)** Kaplan-Meier analysis of disease-free survival curve of PCa patients stratified by PCAT6 expression in TCGA cohort. All experiments were performed in biological triplicate. Statistical analyses were performed by unpaired Student’s *t*-test **(a, b)** and the log-rank test **(c)**. * *p* < 0.05.


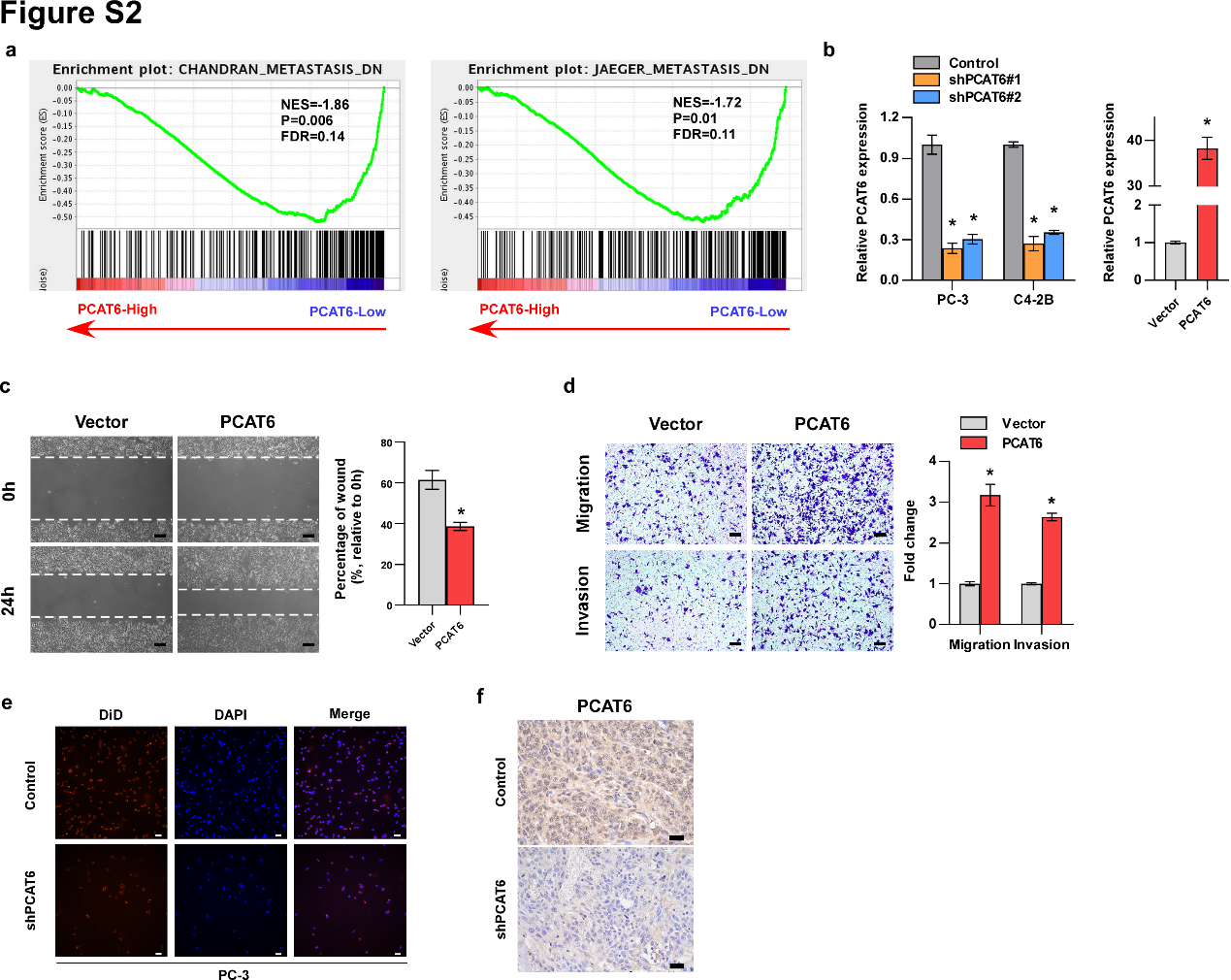
 **Figure S2. PCAT6 promotes PCa cell migration and invasion *in vitro* and bone metastasis *in vivo.***

**(a)** Gene set enrichment analysis (GSEA) revealed that PCAT6 expression was correlated with metastasis signatures. **(b)** RT-qPCR analysis of PCAT6 expression in the indicated cells. Transcript levels were normalized to U6 expression. Error bars represent the mean ± SD of triplicate experiments. **(c)** Representative images of wound-healing assays using 22RV1 cells, showing cell motility after overexpression of *PCAT6* (left panels). Scale bar, 50μm. Histogram analysis of cell migration distances is shown (right panels). Error bars represent the mean ± SD of triplicate experiments. **(d)** Representative images of migration and invasion assays using 22RV1 cells (left panels), showing cell migration and invasion after overexpression of *PCAT6*. Scale bar, 100μm. Histogram analysis of migrated or invaded cell counts is shown (right panels). Error bars represent the mean ± SD of triplicate experiments. **(e)** Representative images of DiD and DAPI staning in the indicated cells. Scar bar, 50μm. **(f)** Representative images of *PCAT6* expression in mice bone metastasis lesions detected by ISH staining. Scar bar, 25μm. All experiments were performed in biological triplicate. Statistical analyses were performed by unpaired Student’s t-test **(b, c, d)**. * *p* < 0.05.


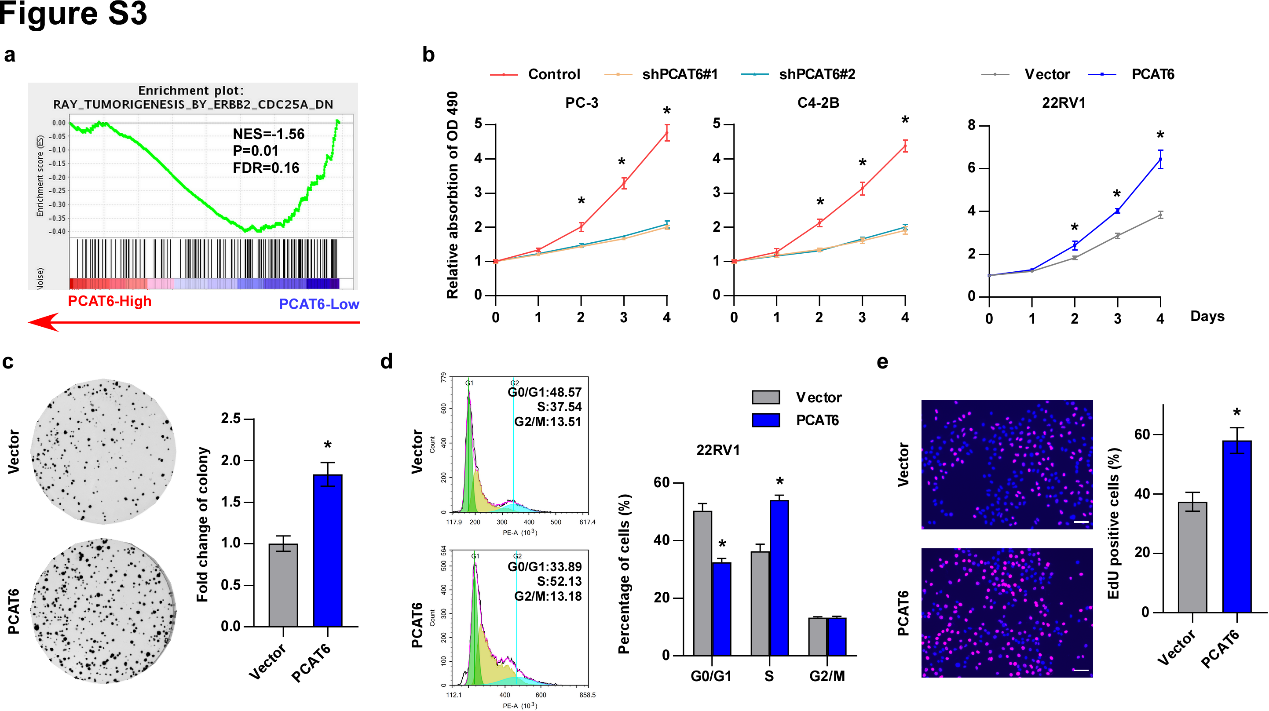
**Figure S3. PCAT6 enhances PCa cell proliferation *in vitro* and tumor growth *in vivo.***

**(a)** Gene set enrichment analysis (GSEA) revealed that PCAT6 expression was correlated with cell proliferation signatures. **(b)** Cell viability was evaluated by MTT assays in the indicated cells. Error bars represent the mean ± SD of triplicate experiments. **(c)** Cell viability was evaluated by colony formation assay in the indicated cells (left panels). Histogram analysis of fold change of colony is shown (right panels). Error bars represent the mean ± SD of triplicate experiments. **(d and e)** Cell populations at different phases were detected by cell cycle analysis **(d)** and EdU **(e)** assays in the indicated cells. PI indicates Propidium Iodide. Scale bar, 100μm. Error bars represent the mean ± SD of triplicate experiments. All experiments were performed in biological triplicate. Statistical analyses were performed by unpaired Student’s t-test **(b, c, d, e)**. * *p* < 0.05.


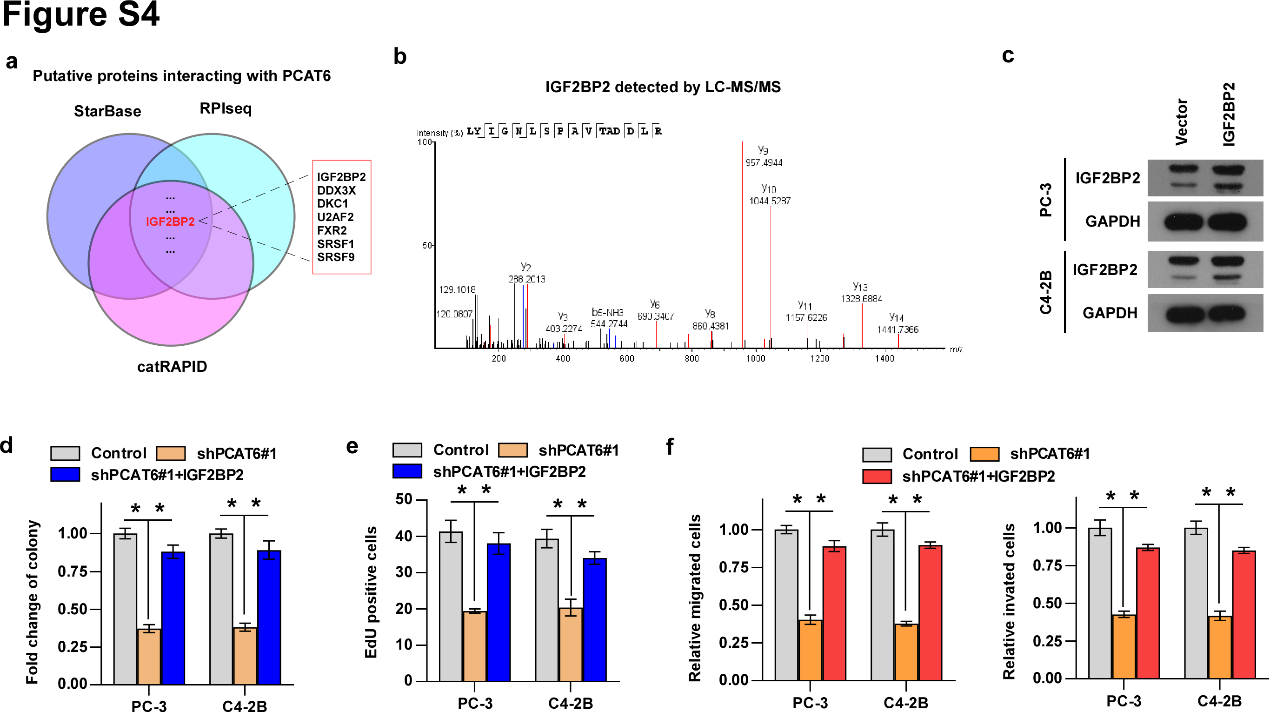
**Figure S4. PCAT6 interacts with IGF2BP2 to play oncogenic roles in PCa.**

**(a)** The putative proteins interacting with PCAT6 predicted by StarBase, RPIseq and catRAPID. **(b)** MS/MS profiles of target band (corresponding peptide sequences of IGF2BP2) retrieved by PCAT6. **(c)** Western blotting analysis of IGF2BP2 expression in the indicated cells. GAPDH served as the loading control. **(d and e)** Cell viability was evaluated by colony formation **(d)** and EdU **(e)** assays in the indicated cells. Error bars represent the mean ± SD of triplicate experiments. **(f)** Transwell assays showing migration and invasion ability of the indicated cells. Error bars represent the mean ± SD of triplicate experiments. All experiments were performed in biological triplicate. Statistical analyses were performed by unpaired Student’s t-test **(d, e, f)**. * *p* < 0.05.

**
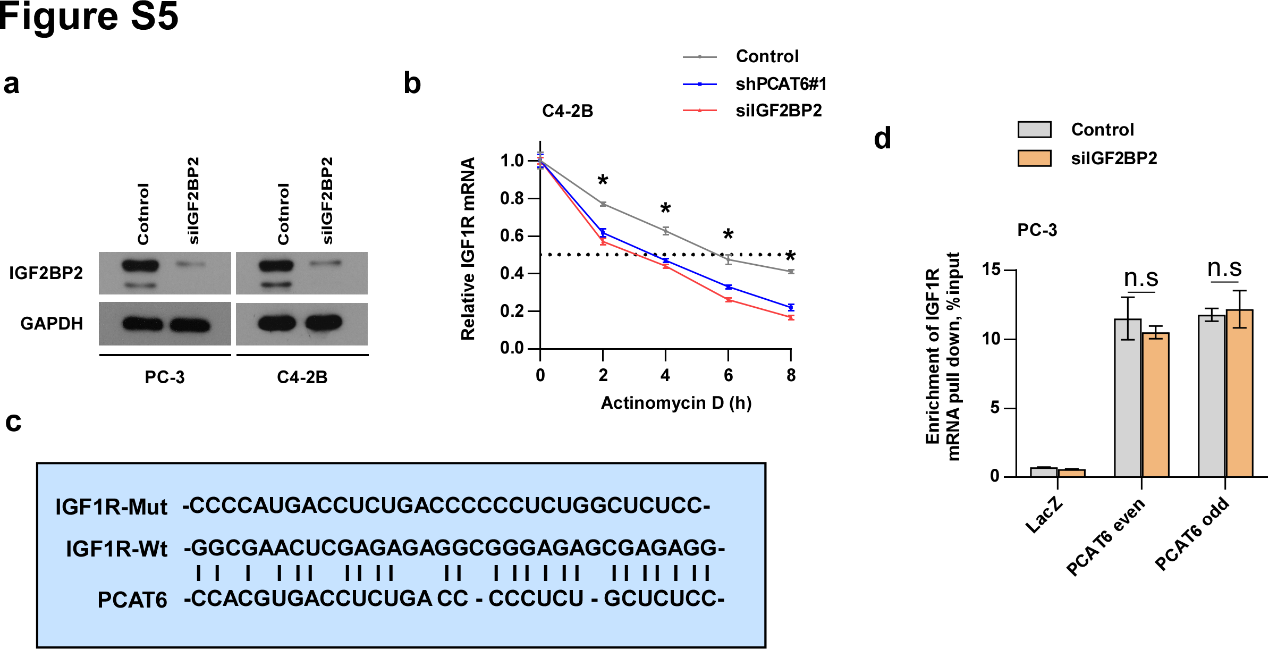
**

**Figure S5. PCAT6 guides IGF2BP2 to stabilize IGF1R mRNA.**

**(a)** Western blotting analysis of IGF2BP2 expression in the indicated cells. GAPDH served as the loading control. **(b)** Control, *PCAT6*-knockdown, or *IGF2BP2*-knockdown C4-2B cells were treated with actinomycin D (5 mg/mL) for the indicated periods. Total RNA was purified and then analyzed using RT-qPCR to examine the mRNA half-lives of *IGF1R*. Error bars represent the mean ± SD of triplicate experiments. **(c)** The binding sites between *PCAT6* and *IGF1R*, and designed mutant IGF1R. **(d)** The enrichment of *IGF1R* mRNA pull down in the indicated group. Error bars represent the mean ± SD of triplicate experiments. All experiments were performed in biological triplicate. Statistical analyses were performed by unpaired Student’s t-test **(b, d)**. n.s indicates no significance.

**
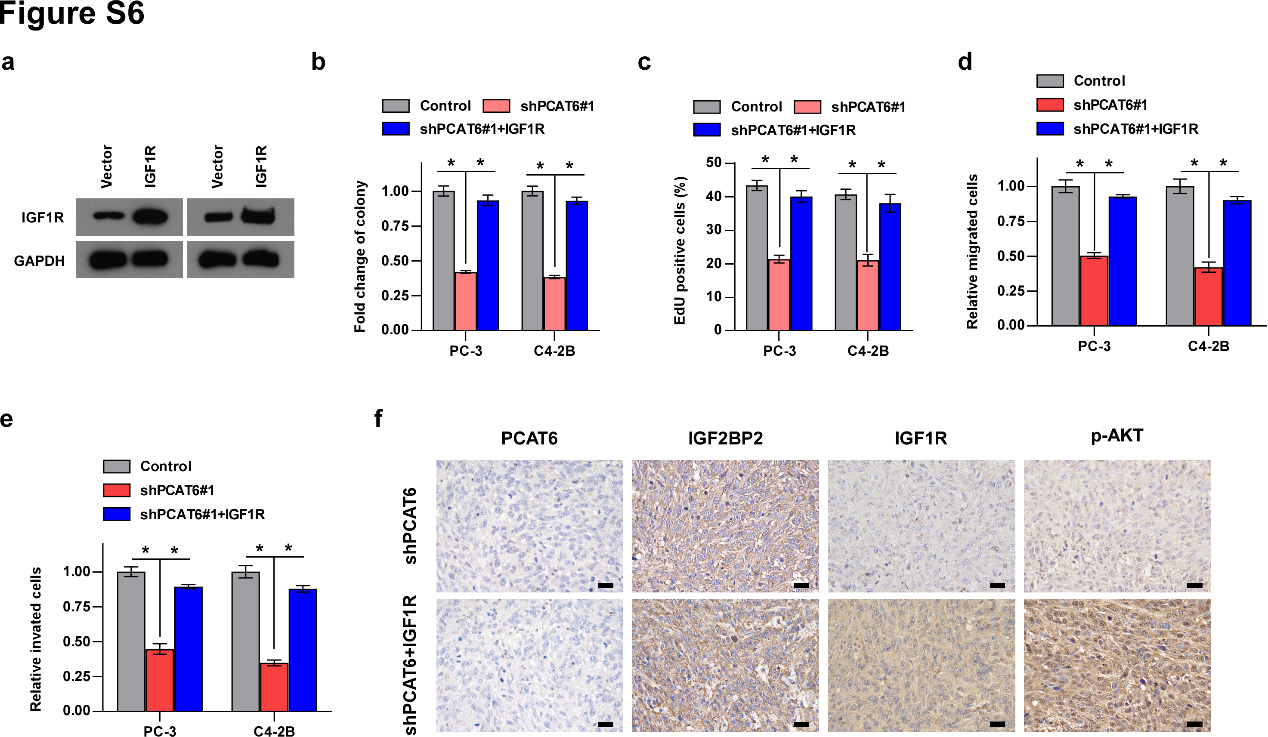
 Figure S6. PCAT6 promotes bone metastasis through IGF1R signaling in PCa.**

**(a)** Western blotting analysis of IGF1R expression in the indicated cells. GAPDH served as the loading control. **(b and c)** Cell viability was evaluated by colony formation **(b)** and EdU **(c)** assays in the indicated cells. Error bars represent the mean ± SD of triplicate experiments. **(d and e)** Transwell assays showing the migration **(d)** and invasion **(e)** ability of the indicated cells. Error bars represent the mean ± SD of triplicate experiments. **(f)** Representative images of *PCAT6*, IGF2BP2, IGF1R and p-AKT expression in mice bone metastasis lesions detected by ISH or IHC staining. Scar bar, 25μm. All experiments were performed in biological triplicate. Statistical analyses were performed by unpaired Student’s t-test **(b, c, d, e)**. * *p* < 0.05.

**
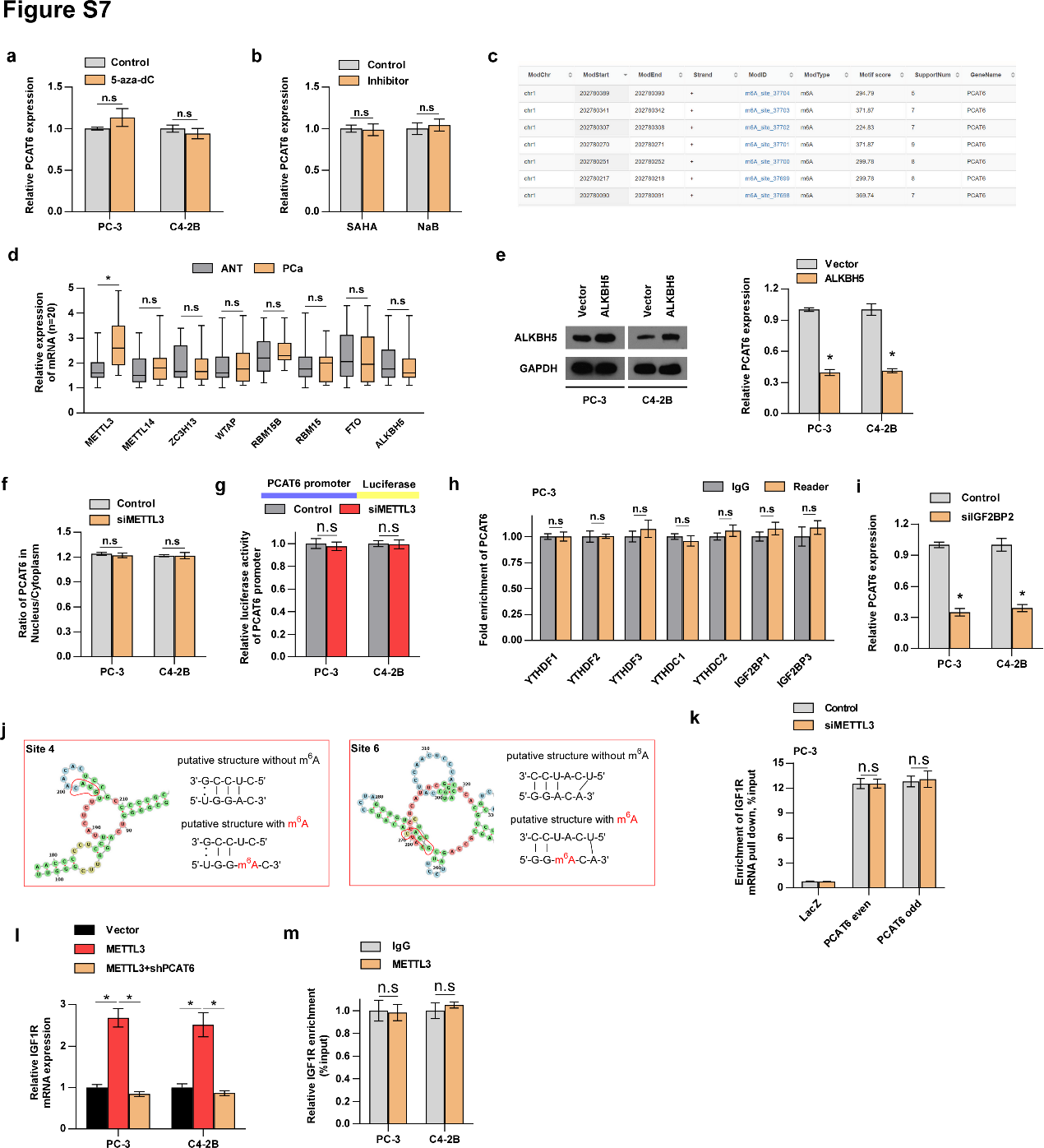
 Figure S7. m^6^A modification contributes to the upregulation of PCAT6 in PCa.**

**(a)** RT-qPCR analysis of PCAT6 expression PC-3 and C4-2B cells with or without treatment of 5-zaz-dC. Transcript levels were normalized to U6 expression. Error bars represent the mean ± SD of triplicate experiments. **(b)** RT-qPCR analysis of PCAT6 expression in PC-3 cells with or without treatment of SAHA or NaB. Transcript levels were normalized to U6 expression. Error bars represent the mean ± SD of triplicate experiments. **(c)** The m^6^A sites in PCAT6 predicted by RMBase. **(d)** RT-qPCR analysis of mRNA expression in ANT (n=20) and PCa (n=20). Transcript levels were normalized to U6 expression. Error bars represent the mean ± SD of triplicate experiments. **(e)** Western blotting analysis of ALKBH5 expression in the indicated cells. GAPDH served as the loading control (left panel). RT-qPCR analysis of PCAT6 expression in the indicated cells (right panel). Transcript levels were normalized to U6 expression. Error bars represent the mean ± SD of triplicate experiments. **(f)** Nuclear–cytoplasmic fractionation assays revealing PCAT6 expression in cytoplasm and nucleus of control or METTL3-knockdown PCa cells. U6 and GAPDH were used as positive controls in the nucleus and cytoplasm, respectively. Error bars represent the mean ± SD of triplicate experiments. **(g)** The luciferase activity of PCAT6 promoter in the indicated group. Error bars represent the mean ± SD of triplicate experiments. **(h)** RIP analysis showing the enrichment of PCAT6 on several proteins in the indicated cells. Transcript levels were normalized to U6 expression. Error bars represent the mean ± SD of triplicate experiments. **(i)** RT-qPCR analysis of PCAT6 expression in the indicated cells. Transcript levels were normalized to U6 expression. Error bars represent the mean ± SD of triplicate experiments. **(j)** The secondary structure of PCAT6 predicted by RNAfold and the putative effect of m^6^A modification on the secondary structure of PCAT6. **(k)** The enrichment of IGF1R mRNA pull down in the indicated group. Error bars represent the mean ± SD of triplicate experiments. **(l)** RT-qPCR analysis of IGF1R expression in the indicated group. Error bars represent the mean ± SD of triplicate experiments. **(m)** Relative IGF1R mRNA enrichment in the indicated group. Error bars represent the mean ± SD of triplicate experiments. All experiments were performed in biological triplicate. Statistical analyses were performed by unpaired Student’s t-test **(a, b, e, f, g, h, i, k, l, m)** and paired Student’s t-test **(d)**. * *p* < 0.05. n.s indicates no significance.


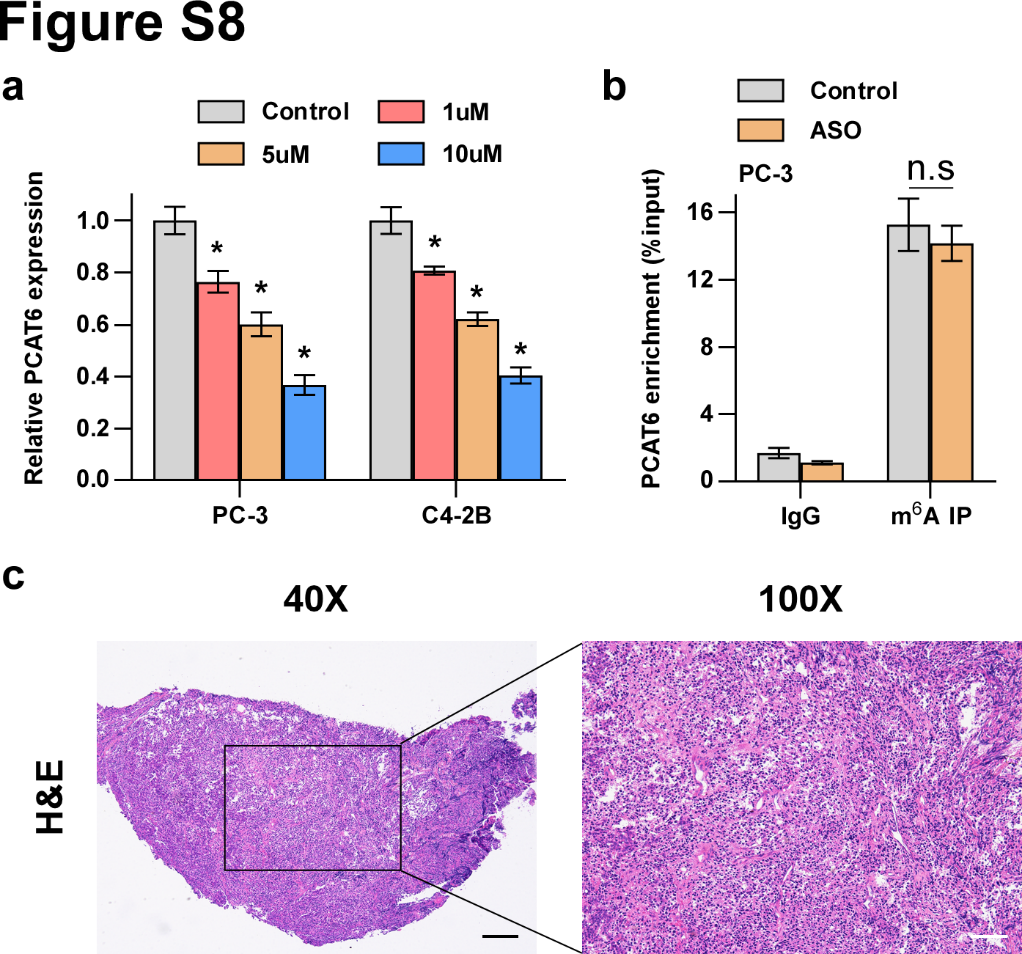


**Figure S8. Clinical relevance of m^6^A/PCAT6/IGF1R axis in PCa.**

**(a)** RT-qPCR analysis of PCAT6 expression in the indicated cells with treatment of different dose ASO targeting PCAT6. Transcript levels were normalized to U6 expression. Error bars represent the mean ± SD of triplicate experiments. **(b)** The m^6^A level of PCAT6 in the indicated group. Error bars represent the mean ± SD of triplicate experiments. **(c)** The histological validation of prostate cancer tissue from the bone. Scar bar: black, 25μm; white, 10μm. All experiments were performed in biological triplicate. Statistical analyses were performed by ANOVA test **(a)** and unpaired Student’s t-test **(b)** . * *p* < 0.05. n.s indicates no significance.

**Supplementary Tables**

**Table S1. List of primers used for RT-qPCR.**

| **Primer Sequence** | |
| --- | --- |
| PCAT6-F | CAGGAACCCCCTCCTTACTC |
| PCAT6-R | CTAGGGATGTGTCCGAAGGA |
| IGF1R-F | CCTGCACAACTCCATCTTCGTG |
| IGF1R-R | CGGTGATGTTGTAGGTGTCTGC |
| GAPDH-F | GTCTCCTCTGACTTCAACAGCG |
| GAPDH-R | ACCACCCTGTTGCTGTAGCCAA |
| U6-F | CTCGCTTCGGCAGCACAT |
| U6-R | TTTGCGTGTCATCCTTGCG |
| METTL3-F | CTATCTCCTGGCACTCGCAAGA |
| METTL3-R | GCTTGAACCGTGCAACCACATC |
| METTL14-F | CTGAAAGTGCCGACAGCATTGG |
| METTL14-R | CTCTCCTTCATCCAGATACTTACG |
| VIRMA-F | TGACCTTGCCTCACCAACTGCA |
| VIRMA-R | AGCAACCTGGTGGTTTGGCTAG |
| WTAP-F | GCAACAACAGCAGGAGTCTGCA |
| WTAP-R | CTGCTGGACTTGCTTGAGGTAC |
| ZC3H13-F | CGGACAGTGATGCCTACAACAG |
| ZC3H13-R | TCTGTGAGGTGCGAGGGACTAA |
| RBM15B-F | TGGTAACCTGGACCACAGCGTA |
| RBM15B-R | GGTTCTGGAACTTGAGGAAGGC |
| RBM15-F | CTTCCCACCTTGTGAGTTCTCC |
| RBM15-R | CTTCTTGTTCTCATACCTAACTCC |
| CBLL1-F | AACAGGATGCCTGCAAAGGCTC |
| CBLL1-R | GGTGTCCAGGAAATCTTCGCTG |
| FTO-F | CCAGAACCTGAGGAGAGAATGG |
| FTO-R | CGATGTCTGTGAGGTCAAACGG |
| ALKBH5-F | CCAGCTATGCTTCAGATCGCCT |
| ALKBH5-R | GGTTCTCTTCCTTGTCCATCTCC |

**Table S2. Relationship between PCAT6 and clinicopathological features in 163 patients with prostate cancer.**

| **Parameters** | **Number of cases** | **PCAT6 expression** | | **P-values** |
| --- | --- | --- | --- | --- |
|  |  | **Low** | **High** |  |
| Age (years) |  |  |  |  |
| <73 | 81 | 49 | 32 |  |
| ≥73 | 82 | 41 | 41 | 0.208 |
| Differentiation |  |  |  |  |
| Well/moderate | 80 | 68 | 12 |  |
| Poor | 83 | 22 | 61 | <0.05* |
| Serum PSA |  |  |  |  |
| <67.9 | 81 | 66 | 15 | <0.05* |
| ≥67.9 | 82 | 24 | 58 |  |
| Gleason grade |  |  |  |  |
| ≤7 | 88 | 70 | 18 |  |
| >7 | 75 | 20 | 55 | <0.05* |
| BM status |  |  |  |  |
| nBM | 122 | 79 | 43 |  |
| BM | 41 | 11 | 30 | <0.05* |

**Abbreviation: PSA, Prostate-specific Antigen; SD, Standard Deviation; BM, Bone Metastasis; n-BM: Non-bone Metastasis.**

**Table S3. Gene Symbols of 100 mRNAs most significantly bound by IGF2BP2 in 293 cells**

(The genes shown in yellow were involved in prostate cancer progression in previous studies.)

NUCKS1

SRSF1

MED13

PCBP2

PEG10

MYC

TAOK1

NUDT21

TOMM20

PTP4A1

NRAS

CCT2

TMED2

CCNG1

ADNP

ICMT

CBX5

PTPLAD1

PROSER1

JMY

RPL15

CLIC4

ANKRD40

UHMK1

XIST

YARS

UBXN2B

CDK6

TMBIM6

SRCAP

IGF1R

FHL1

NUFIP2

KLHL15

PRRC2C

CERS2

PGAM5

CANX

QSER1

MLL5

SRRM2

SP1

LCOR

PPPDE1

SERBP1

SCD

LARP1

TEX261

AKT3

ZNF146

SLC7A1

GRPEL2

MAP1B

H3F3B

C5orf51

HNRNPUL1

TMPO

ARPP19

PAICS

ERH

MDM2

TUBB

PURB

NR3C1

TUG1

SLC38A2

WNK1

NOTCH2

MUC16

CALU

RMND5A

LRRC58

ZNF664

CAPZA1

FSTL1

RLIM

CCND2

SCML1

YWHAE

ANP32A

EIF1

SLC38A1

ZNF829

TOR1AIP2

GLO1

GNG12

FAM208B

CSNK2A1

MKI67

ARF1

BCLAF1

JAGN1

EEF2

HSP90AA1

RPL18

KPNA6

HNRNPU

MAPK1

AP3M1

**Table S4.** **Clinicopathological features of 163 prostate cancer patients**

| **Parameters** | **Number of cases** |
| --- | --- |
| Age (years) |  |
| <73 | 81 |
| ≥73 | 82 |
| Median | 73 |
| Differentiation |  |
| Well/moderate | 80 |
| Poor | 83 |
| Serum PSA at diagnosis, ng/ml |  |
| <67.9 | 81 |
| ≥67.9 | 82 |
| Median | 67.9 |
| SD | 154.4 |
| Mean | 97.7 |
| Gleason grade |  |
| ≤7 | 88 |
| >7 | 75 |
| PCAT6 expression (SI) |  |
| ≤4 | 90 |
| >4 | 73 |
| BM status |  |
| nBM | 122 |
| BM | 41 |

**Abbreviation: PSA, Prostate-specific Antigen; SD, Standard Deviation; BM, Bone Metastasis; n-BM: Non-bone Metastasis.**

**Table S5. The sequences of probes used in RNA pulldown assay**

| **Probe** | **Sequence (5’-3’)** | | **Label** |
| --- | --- | --- | --- |
| PCAT6-even-1 | GTTGCCCAGGTCCGGCCTCC | 3’-biotin | |
| PCAT6-even-2 | TGACCCCGCCTAGGCCTCGC | | 3’-biotin |
| PCAT6-even-3 | GCGGAGTGTTGTCCAAGAGT | | 3’-biotin |
| PCAT6-even-4 | GATGGACCGAATGAGGATGG | | 3’-biotin |
| PCAT6-even-5 | GCGAGGAGCGCCTCATCACC | | 3’-biotin |
| PCAT6-even-6 | ACGAGAGGATCCACCGGGTT | | 3’-biotin |
| PCAT6-odd-1 | GCTCCGGCCCCGGCACCTCC | | 3’-biotin |
| PCAT6-odd-2 | CGCCTGGGCTGCAGCTCCGC | | 3’-biotin |
| PCAT6-odd-3 | TGCACAAATGAGGGCGGTTT | | 3’-biotin |
| PCAT6-odd-4 | CTAGGGGCGAAGGCTGCAGA | | 3’-biotin |
| PCAT6-odd-5 | GGTAGAAGCACGAGCAAGGC | | 3’-biotin |
| PCAT6-odd-6 | CATCTGACAGCAAACATTCC | | 3’-biotin |
| LacZ-1 | CCAGTGAATCCGTAATCATG | | 3’-biotin |
| LacZ-2 | ATTAAGTTGGGTAACGCCAG | | 3’-biotin |
| LacZ-3 | AATGTGAGCGAGTAACAACC | | 3’-biotin |
| LacZ-4 | AATAATTCGCGTCTGGCCTT | | 3’-biotin |
| LacZ-5 | AGATGAAACGCCGAGTTAAC | | 3’-biotin |
| LacZ-6 | TCACGACGTTGTAAAACGAC | | 3’-biotin |

**Supplementary Methods**

**Cell lines and culture**

The human prostate cancer cell lines, PC-3, C4-2B, 22RV1, DU145, VCaP and LNCaP and the normal prostate cell line RWPE-1 were used in this study. C4-2B cells were obtained from the MD Anderson Cancer Center, and the remaining cell lines were purchased from the Shanghai Chinese Academy of Sciences cell bank (China). All cell lines were cultured with the recommended medium supplemented with 10% fetal bovine serum (Life Technologies, USA), streptomycin (100 mg/ml), and penicillin G (100 U/ml) in a humidified atmosphere of 5% CO_2_ at 37 °C.

**Human samples and tissues**

A total of 122 primary PCa tissues without bone metastasis, 41 primary PCa tissues with bone metastasis, 26 adjacent normal prostate tissues and 18 metastatic PCa tissues from bone were obtained during surgery or needle biopsy at The First People’s Hospital of Guangzhou City (Guangzhou, China) between January 2003 and October 2019. For bone metastasis tissues, we firstly obtained the tissues with high tumor content according to our experience during operation. Next, slides were cut from frozen tissue and examined by the study pathologists (D.H.) to select high-density cancer foci, ensure high purity of cancer tissue and avoid regions of necrosis or high stromal content. Tissue specimens with more than 80% cancerous tissue were used for the further study. The histological validation was provided in Supplementary Fig.S8c. For the use of these clinical materials for research purposes, prior patient consent and approval from the Institutional Research Ethics Committee were obtained. The clinicopathological features of the patients are summarized in Supplementary Table S4. The median *PCAT6* expression in PCa tissues was used to stratify the high and low expression of *PCAT6*.

**Plasmid, small interfering RNA and transfection**

Human PCAT6, IGF2BP2 and IGF1R cDNA was PCR-amplified and cloned into the pMSCV-puro-retro vector (Clontech, PaloAlto, CA, USA). Two shRNAs against PCAT6 (targeting sequences: shPCAT6#1, 5′-GGTGTCTCCATCCTCATTC-3′; shPCAT6#2, 5′-CTCCCAGACCTCACGTCAA-3′) were cloned into the pLKO.1-puro lentivirus vector (Addgene, Watertown, MA, USA). Viral production and infection were performed as previously described[^1^](#_ENREF_1). Briefly, transfection of plasmids was performed using Lipofectamine 3000 reagent (Invitrogen, Carlsbad, California, USA) based on the manufacturer’s instructions. Cells (2×10^5^) were cultured and infected using a virus produced by pMSCV-puro-PCAT6, -IGF2BP2 and -IGF1R and pLKO.1-puro-shPCAT6#1 and #2 for 3 days. Stable cell lines expressing PCAT6, IGF2BP2, IGF1R and shPCAT6 were selected with 0.5μg/mL puromycin for 7 days. The transfection efficiency was determined by RT-qPCR or western blotting assays. Small interfering RNAs (siRNAs) targeting IGF2BP2 (targeting sequences: 5’-CATGCCGCATGATTCTTGA-3’) and METTL3 (targeting sequences: 5’-GCACTTGGATCTACGGAAT-3’) were synthesized by RiboBio (Guangzhou, China).

**RNA fluorescence in situ hybridization (RNA FISH) and subcellular fractionation assays**

The Cy3-labeled PCAT6 probe was purchased from RiboBio (Guangzhou, China). RNA FISH was performed using a FISH Kit (RiboBio, Guangzhou, China) according to the manufacturer’s instructions. A nucleus and cytoplasm segmentation PARIS^TM^ Kit (Ambion, Austin, TX, USA) was used to separate the nuclear and cytoplasmic fractions of cells following the manufacturer’s instructions.

**The Cancer Genome Atlas (TCGA),** **Gene Expression Omnibus (GEO) and Gene Set Enrichment Analysis (GSEA)**

LncRNA and mRNA expression profiles analyzed in this study were downloaded from TCGA (<https://gdc.cancer.gov/>) or GEO (<https://www.ncbi.nlm.nih.gov/geo/>). GSEA (<https://www.gsea-msigdb.org/gsea/index.jsp>) based on TCGA dataset was performed in accordance with previous description[^2^](#_ENREF_2). The samples are grouped according to the median of PCAT6 expression as the cutoff.

**Migration, invasion and wound healing assays**

Migration and invasion assays were performed using a Transwell chamber consisting of 8-mm membrane filter inserts (Corning, NY, USA) with or without Matrigel (BD Biosciences, Franklin Lakes, NJ, USA) coating. The detailed information is described in our previous study[^3^](#_ENREF_3). Briefly, PCa cells (2 × 10^4^) were seeded into the upper compartment of Transwell chambers, and the lower chamber of the Transwell was filled with complete media supplemented with 10% FBS. After incubation for 24h, cells migrating or invading to the bottom side of the chamber were fixed with methanol and stained with crystal violet. Three fields in one membrane (three membranes were included in every group) were selected to count migrated/invaded cells under the microscope (x100) and the average was taken as the final number of migrated/invaded cells in the indicated group. The number of migrated/invaded cells in control group was selected as the control (=1) and the fold change was calculated as the ratio of the number of migrated/invaded cells in every group relative to that in the corresponding control group. PCa cells (1×10^6^) were seeded in 6-well plates overnight, and wounds were made using a 100 μl plastic pipette tip. The size of the wound was measured after 24 h after wound formation, and the wound was imaged.

**Western blotting**

Western blotting was carried out as previously reported[^1^](#_ENREF_1). Antibodies against IGF1R, p-AKT (ser473) and p65 were provided by Cell Signaling Technology (Danvers, MA, USA), and antibodies against METTL3 and IGF2BP2 were purchased from Abcam (Cambridge, UK) and Proteintech (Wuhan, China), respectively. Anti-GAPDH (Proteintech, Wuhan, China) and anti-p84 (Invitrogen, Carlsbad, CA, USA) antibodies were used as the loading controls.

**Luciferase reporter assay**

The luciferase reporter assay was performed with the Dual-Luciferase Reporter Assay System (Promega) according to the manufacturer’s protocol. The pmiGLO-based luciferase vector fused or not fused to the wild-type or mutated IGF1R were transfected into cells using the Lipofectamine 3000 reagent (Invitrogen). Twenty-four hours after transfection, luciferase and Renilla signals were measured using the Dual Luciferase Reporter Assay Kit (Promega), according to the manufacturer’s instructions.

**RNA pull-down and RNA immunoprecipitation (RIP) assay**

*PCAT6* full-length sense, antisense, and serial deletion sequences were prepared via *in vitro* transcription using a Transcript Aid T7 High Yield Transcription Kit (Thermo Scientific, Waltham, MA, USA). The RNA pull-down assay was performed using a Magnetic RNA-Protein Pull-down Kit (Thermo Scientific, Waltham, MA, USA) according to the manufacturer’s instructions and as described previously[^4^](#_ENREF_4). The method to pull down RNAs interacting with *PCAT6* is shown below. 3’end biotin-labeled oligonucleotide probes targeting *PCAT6* transcript were synthesized by Sangon. Twelve probes targeting PCAT6 were designed and numbered. To eliminate nonspecific signals, six probes with an odd number were assigned into odd group and six probes with an even number were assigned into even group. The probe set against LacZ was used as a negative control. The sequences of probes are shown in Supplementary Table S5. The detailed procedure was described in a previous study[^5^](#_ENREF_5). The RIP assay was carried out using the Magna RIP RNA-Binding Protein Immunoprecipitation Kit (Millipore, Burlington, MA, USA) according to a previous study[^6^](#_ENREF_6). In brief, the cells were lysed in a RIP lysis buffer, and magnetic beads were conjugated with a human antibody against *IGF2BP2* (Proteintech, Wuhan, China) or m^6^A (Synaptic Systems) with normal mouse IgG antibody (Millipore, Burlington, MA, USA) as a negative control. Subsequently, the obtained RNA was assessed by RT-qPCR and normalized to the input.

1. Ren D, Dai Y, Yang Q, Zhang X, Guo W, Ye L, Huang S, Chen X, Lai Y, Du H, Lin C, Peng X and Song L. Wnt5a induces and maintains prostate cancer cells dormancy in bone. *The Journal of experimental medicine*. 2019;216:428-449.

2. Lang C, Dai Y, Wu Z, Yang Q, He S, Zhang X, Guo W, Lai Y, Du H, Wang H, Ren D and Peng X. SMAD3/SP1 complex-mediated constitutive active loop between lncRNA PCAT7 and TGF-β signaling promotes prostate cancer bone metastasis. *Molecular oncology*. 2020;14:808-828.

3. Ren D, Yang Q, Dai Y, Guo W, Du H, Song L and Peng X. Oncogenic miR-210-3p promotes prostate cancer cell EMT and bone metastasis via NF-kappaB signaling pathway. *Molecular cancer*. 2017;16:117.

4. Gu P, Chen X, Xie R, Xie W, Huang L, Dong W, Han J, Liu X, Shen J, Huang J and Lin T. A novel AR translational regulator lncRNA LBCS inhibits castration resistance of prostate cancer. *Molecular cancer*. 2019;18:109.

5. Chen Z, Chen X, Xie R, Huang M, Dong W, Han J, Zhang J, Zhou Q, Li H, Huang J and Lin T. DANCR Promotes Metastasis and Proliferation in Bladder Cancer Cells by Enhancing IL-11-STAT3 Signaling and CCND1 Expression. *Mol Ther*. 2019;27:326-341.

6. Li T, Hu P-S, Zuo Z, Lin J-F, Li X, Wu Q-N, Chen Z-H, Zeng Z-L, Wang F, Zheng J, Chen D, Li B, Kang T-B, Xie D, Lin D, Ju H-Q and Xu R-H. METTL3 facilitates tumor progression via an m6A-IGF2BP2-dependent mechanism in colorectal carcinoma. *Molecular cancer*. 2019;18.
